# AAV vectors for specific and efficient gene expression in microglia

**DOI:** 10.1016/j.crmeth.2025.101116

**Published:** 2025-07-30

**Authors:** Ryo Aoki, Ayumu Konno, Nobutake Hosoi, Hayato Kawabata, Hirokazu Hirai

**Affiliations:** 1 Department of Neurophysiology & Neural Repair, Gunma University Graduate School of Medicine, Maebashi, Gunma 371-8511, Japan; 2 Viral Vector Core, Gunma University, Initiative for Advanced Research, Maebashi, Gunma 371-8511, Japan

**Keywords:** microglia, AAV, adeno-associated virus, Iba1, microRNA, RNA interference, WPRE, cerebral cortex, Ca^2+^ imaging, GCaMP

## Abstract

Microglia are crucial targets for therapeutic interventions in diseases like Alzheimer’s and stroke, but efficient gene delivery to these immune cells is challenging. We developed an adeno-associated virus (AAV) vector that achieves specific and efficient gene delivery to microglia. This vector incorporates the mIba1 promoter, GFP, miRNA target sequences (miR.Ts), WPRE, and poly(A) signal. Positioning miR.Ts on both sides of WPRE significantly suppressed non-microglial expression, achieving over 90% specificity and more than 60% efficiency in microglia-specific gene expression 3 weeks post-administration. Additionally, this vector enabled GCaMP expression, facilitating real-time calcium dynamics monitoring in microglial processes. Using a blood-brain barrier-penetrant AAV-9P31 capsid variant, intravenous administration resulted in broad and selective microglial GFP expression across the brain. These results establish our AAV vector as a versatile tool for long-term, highly specific, and efficient gene expression in microglia, advancing microglial research and potential therapeutic applications.

## Highlights

• AAV vector enables specific and efficient gene expression in cortical microglia
• Placing miRNA targets flanking WPRE enhances microglial specificity and efficiency
• Vector enables *in vivo* calcium imaging and dynamic analysis of microglial processes
• Systemic delivery using 9P31 capsid achieves broad microglial targeting

## Motivation

Selective and efficient gene delivery to microglia has long posed a challenge due to their antiviral defense properties and low permissiveness to viral vectors. Our previous strategy employing a microglia-selective promoter and microRNA detargeting has demonstrated partial success, but failed to achieve robust specificity and efficiency, particularly in the cerebral cortex. We aimed to address these limitations by systematically optimizing the regulatory architecture of AAV vectors, focusing on the spatial arrangement of microRNA target sequences relative to WPRE. Our goal was to develop a robust, selective gene delivery tool applicable to *in vivo* microglial research and potential therapeutic interventions.

## Graphical abstract

**Figure:**
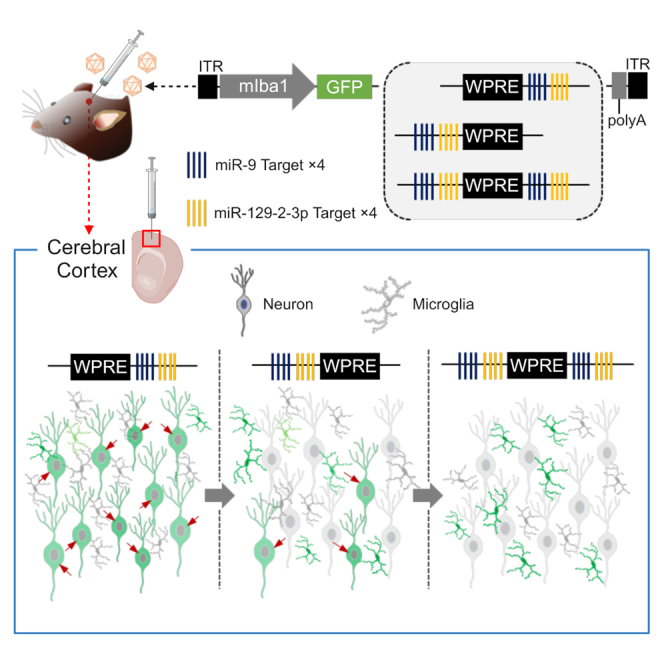

## Teaser

Aoki et al. develop an AAV vector for highly specific and efficient gene expression in cortical microglia. By flanking WPRE with microRNA target sequences, they achieve >90% specificity and enable functional imaging of microglial Ca^2+^ dynamics. This vector also permits systemic delivery, facilitating broad access to microglia *in vivo*.

## Introduction

Microglia, the resident immune cells of the central nervous system (CNS), originate from progenitor cells in the fetal yolk sac and play essential roles in monitoring and regulating neuronal activity.^1^ By extending their processes, microglia make dynamic contacts with synapses and axons, contributing to the maintenance of neuronal function.^2^^,^^3^ In pathological conditions, microglia become activated, migrating to lesions,^4^ phagocytosing damaged cells,^5^^,^^6^ and releasing various humoral factors.^7^^,^^8^ Microglial activation has been implicated in the pathogenesis of several CNS diseases, including Alzheimer’s disease^9^^,^^10^ and multiple sclerosis,^11^^,^^12^ making these cells attractive therapeutic targets.

Given their critical role in brain homeostasis and disease, microglia have become important targets for genetic manipulation. However, selectively expressing genes in microglia using viral vectors has proven challenging, as microglia are involved in antiviral defense mechanisms within the CNS. In 2013, Jakobsson and co-workers successfully achieved selective transgene expression in microglia by using lentiviral vectors with the phosphoglycerate kinase (PGK) promoter and microRNA-9-target (miR-9.T) sequences.^13^ Because miR-9 is highly expressed in neurons and astrocytes but not in microglia, the presence of miR-9.T led to degradation of transgene mRNA in non-microglial cells. As a result, selective gene expression was achieved in microglia, with over 70% of transgene-expressing cells being microglia in the rat striatum. However, when the PGK promoter was replaced by the stronger cytomegalovirus promoter, transgene expression leaked into neurons and astrocytes.^14^

To address these issues, we previously developed an adeno-associated virus serotype 9 (AAV9) vector that targets microglia. This vector combines a mouse-derived microglia/macrophage-specific ionized calcium-binding adaptor molecule 1 (mIba1) promoter with miR-9.T and miR-129-2-3p.T sequences.^15^ These microRNA targets (miR.T), expressed in neurons but not in microglia, allowed for selective transgene expression in microglia within the striatum and cerebellum following brain parenchymal injection. However, while transgene expression in non-target neurons of the cerebral cortex was not prominent 1 week after injection, by 3 weeks, a substantial number of neurons exhibited strong transgene expression. This highlighted a major unresolved challenge in achieving microglia-specific expression in the cerebral cortex.^15^

Recent efforts to enhance microglial targeting have included the development of AAV capsid variants. Lin et al. identified two AAV9 capsid mutants, AAV-MG1.1 and AAV-MG1.2, through *in vivo* screening.^16^ Although these capsid mutants were able to transduce microglia, they also transduced neurons and astrocytes, limiting their specificity. Similarly, Young et al. developed AAV capsid mutants capable of crossing the blood-brain barrier (BBB) and efficiently transducing microglia.^17^ Although GFP expression was observed very efficiently in microglia, these mutants also induced GFP expression in neurons, oligodendrocytes, and astrocytes, highlighting the persistent challenge of achieving microglia-specific gene expression in the cerebral cortex.

In this study, we aimed to overcome these limitations and, consequently, to develop AAV vectors with both high specificity and efficiency in transgene expression targeting cortical microglia. Our goal was to create a tool for long-term, microglia-specific gene expression in the cerebral cortex that could be used to study microglial function and serve as a platform for potential therapeutic interventions targeting microglia in neuropsychiatric disorders.

## Results

### The addition of miR-708-5p.T×3 to the existing miR-9.T and miR-129-2-3p.T did not enhance neuron detargeting

**Figure 1:**
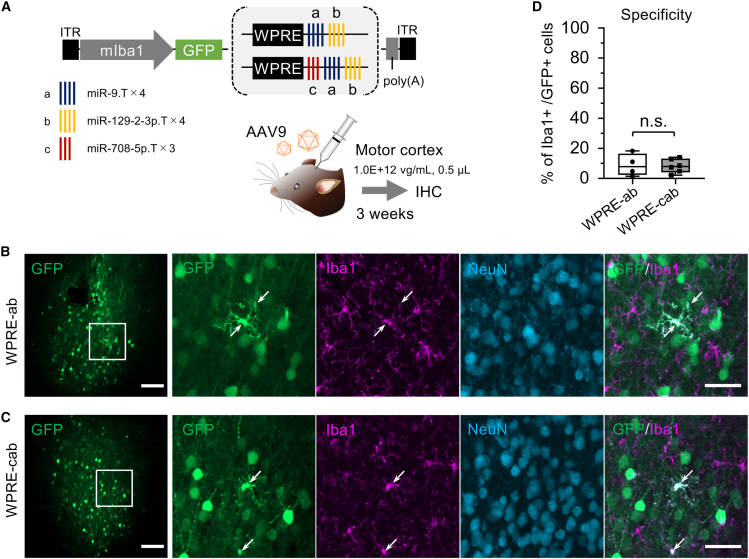
Addition of triplet miR-708-5p.T fails to enhance microglia detargeting (A) Schematic depicting the AAV genome comprising microRNA target sequences: the quadruplet or triplet microRNA target sequences miR-9.T×4, miR-129-2-3p.T×4, and miR-708-5p.T×3, abbreviated as “a,” “b,” and “c,” respectively. Triplet miR-708-5p.T was inserted between WPRE and the quadruplet miR-9.T in the genome of a previously reported microglia-targeting AAV harboring WPRE-ab. The genomes were packaged with the AAV9 capsid. Six- to eight-week-old C57BL/6J mice received an injection of either one of the AAV vectors (1.0E+12 vg/mL, 0.5 μL) into the motor cortex. Three weeks after injection, mice were euthanized, and cerebral sections were prepared and analyzed by immunohistochemistry. (B and C) Confocal laser-scanning microscopy of cerebral sections injected with a control AAV carrying WPRE-ab (B) and those injected with AAV carrying WPRE-cab (C). The sections were triple immunostained for GFP, Iba1, and NeuN. Arrows indicate GFP-expressing Iba1-positive microglia. Scale bars, 100 μm (left, low magnification) and 20 μm (right, enlarged images). (D) Summarized graph showing the specificity of microglia transduction in the two mouse groups. n.s., not significant (*p* > 0.05) by unpaired t tests (*n* = 4 hemispheres for WPRE-ab, *n* = 6 hemispheres for WPRE-cab). The box-and-whisker plots depict the median (centerlines), 25th and 75th percentiles (bounds of the box), and minimum/maximum values (whiskers).

In our previous study, we found that inserting quadruplet miR-9.T downstream of the woodchuck hepatitis virus posttranscriptional regulatory element (WPRE) in the AAV.mIba1.GFP.WPRE construct improved microglial specificity of GFP expression in the mouse cerebral cortex.^15^ Additionally, the inclusion of quadruplet miR-129-2-3p.T further enhanced microglial specificity.^15^ Since miR-708-5p is expressed in neurons but not in microglia,^18^ we investigated whether adding miR-708-5p.T to the AAV.mIba1.WPRE.GFP.miR-9.T.miR-129-2-3p.T construct could improve neuron detargeting. For simplicity, the miR-9.T, miR-129-2-3p.T, and miR-708-5p.T sequences are abbreviated as “a,” “b,” and “c,” respectively (Figure 1A).

We injected AAV.mIba1.GFP.WPRE-cab (1.0E+12 vg/mL, 0.5 μL) or AAV.mIba1.GFP.WPRE-ab into the motor cortex of mice (*n* = 6 for WPRE-cab, *n* = 4 for WPRE-ab) and analyzed brain sections 3 weeks later. Immunohistochemistry revealed that, similar to the cortex injected with AAV.mIba1.GFP.WPRE-ab, numerous GFP-positive neurons co-labeled with NeuN were also observed in the cerebral cortex injected with AAV.mIba1.GFP.WPRE-cab (Figures 1B and 1C). Quantitative analysis confirmed that the addition of miR-708-5p.T did not significantly improve neuron detargeting, as the microglial specificity of GFP expression was similar between WPRE-cab (8.2% ± 4.2%, *n* = 6 hemispheres) and WPRE-ab (8.8% ± 6.4%, *n* = 4 hemispheres) (Figure 1D).

### Placing miR.T sequences upstream of WPRE significantly enhances neuron detargeting

During AAV transduction in neurons, the viral genome is transported to the nucleus, where mRNA is transcribed. It has been shown that miR.T-containing mRNA is cleaved between the 9th and 10th base pairs downstream of the 5′ side of the miR.T sequence.^19^^,^^20^ When miR.T sequences are positioned downstream of WPRE (AAV.mIba1.WPRE-ab), the mRNA is cleaved at the site downstream of WPRE, resulting in an mRNA consisting of GFP and WPRE (Figures S1A and S1B). WPRE stabilizes the mRNA, which enhances protein expression levels, independent of the transgene or promoter.^21^ Consequently, it is hypothesized that, even though the resulting GFP-WPRE mRNA lacks a polyadenylation (poly(A)) signal, it is still translated into GFP protein due to the stabilizing effects of WPRE. In contrast, when the miR.T sequence is inserted between GFP and WPRE, the mRNA is cleaved between these two elements, resulting in an mRNA consisting only of GFP (Figure S1C). However, this truncated GFP mRNA lacks both WPRE and the poly(A) signal, leading to degradation, and it is expected that no GFP protein will be produced from this mRNA.

To confirm whether GFP protein expression occurs in the presence of WPRE without poly(A), but not in its absence, we prepared AAVs with the following sequences in the AAV genome: mIba1.GFP.WPRE.poly(A), mIba1.GFP.WPRE-ab.poly(A), mIba1.GFP.WPRE, and mIba1.GFP (Figure S1D). These AAVs were injected into the motor cortex of mice (1.0E+12 vg/mL, 1.0 μL), and GFP fluorescence was observed 3 weeks post-injection. As a result, strong GFP expression was observed in mIba1.GFP.WPRE.poly(A), while weak but comparable GFP expression was detected in mIba1.GFP.WPRE-ab.poly(A) and mIba1.GFP.WPRE (Figures S1E–S1G). It was hypothesized that GFP.WPRE-ab.poly(A) mRNA is cleaved at the ab (miR.T) site, producing GFP-WPRE mRNA. Additionally, it was found that GFP protein production was nearly absent when both WPRE and poly(A) were missing (Figure S1H). These results suggested that placing miR.T between GFP and WPRE enhances the suppression of GFP protein expression due to mRNA cleavage between GFP and WPRE (Figure S1C).

**Figure 2:**
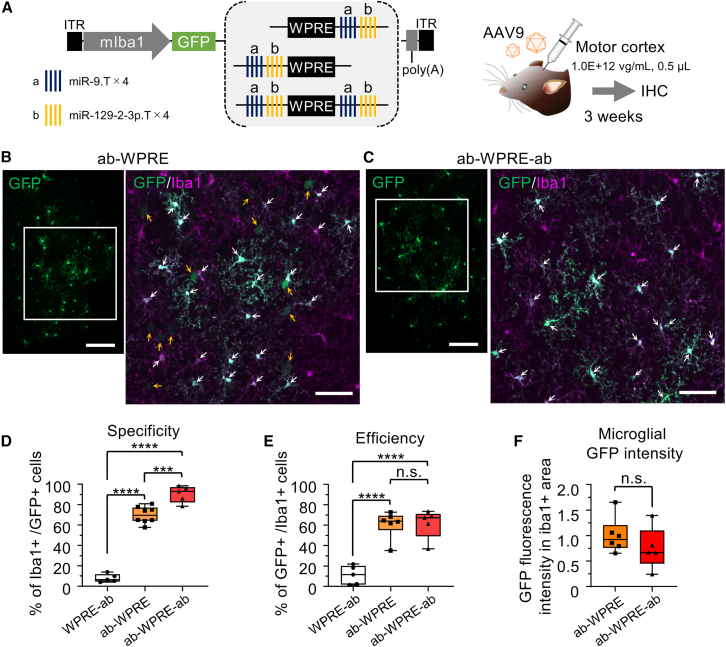
Significant enhancement of specificity and efficiency of GFP expression in microglia by placing the miR.T sequence upstream of WPRE (A) Schematic depicting the AAV genome with microRNA target sequences placed on the 3′ side (control), 5′ side, or both sides of WPRE. Mice received an injection of either one of the AAV9 vectors (1.0E+12 vg/mL, 0.5 μL) into the motor cortex and were euthanized for immunohistochemistry 3 weeks post-injection. (B and C) Confocal microscopy of cerebral sections immunostained from mice injected with AAV.mIba1.GFP.ab-WPRE (ab-WPRE) (B) and those injected with AAV.mIba1.GFP.ab-WPRE-ab (ab-WPRE-ab) (C). For the left and right panels, the left side shows low magnification images of GFP immunostaining, while the right side shows high-magnification images of GFP and Iba1 immunostaining, which are enlarged views of the boxed areas in the left images. White and yellow arrows indicate transduced microglia and neurons, respectively. Scale bars, 100 μm (left, low magnification) and 50 μm (right, enlarged images). (D and E) Summarized graphs showing specificity (*n* = 5 hemispheres for WPRE-ab, *n* = 8 hemispheres for ab-WPRE, *n* = 5 hemispheres for ab-WPRE-ab) (D) and efficiency (*n* = 5 hemispheres for WPRE-ab, *n* = 6 hemispheres for ab-WPRE, *n* = 5 hemispheres for ab-WPRE-ab) (E) of microglia transduction in the three mouse groups. Efficiency of microglia transduction was calculated as the number of GFP- and Iba1-double-positive cells divided by the number of Iba1-immunolabeled microglia within a 320 × 320 μm area. ∗∗∗*p* < 0.001, ∗∗∗∗*p* < 0.0001; n.s., not significant (*p* > 0.05) by Bonferroni’s multiple comparisons test following one-way ANOVA. (F) A graph comparing GFP fluorescence values in the Iba1+ area of mice injected with AAV carrying ab-WPRE (*n* = 6 hemispheres) or AAV carrying ab-WPRE-ab (*n* = 5 hemispheres). The total GFP fluorescence intensity in a 320 × 320 μm Iba1+ area was measured, setting the value for AAV carrying ab-WPRE at 1. n.s., not significant (*p* > 0.05) by unpaired t tests. (D–F) The box-and-whisker plots depict the median (centerlines), 25th and 75th percentiles (bounds of the box), and minimum/maximum values (whiskers). See also Figures S1–S4.

To test this, we created two AAV vectors: AAV.mIba1.GFP.ab-WPRE and AAV.mIba1.GFP.ab-WPRE-ab, where the miR.T sequences were positioned upstream and on both sides of WPRE, respectively (Figure 2A). These vectors were injected into the motor cortex of mice. Immunohistochemistry revealed a marked reduction in the number of GFP^+^ neurons with both constructs (Figures 2B and 2C). Quantitative analysis showed that the microglial specificity of GFP expression was significantly higher in both AAV vectors with miR.T sequences upstream of WPRE (ab-WPRE: 69.9% ± 7.6%, *n* = 8 hemispheres) and those with miR.T sequences on both sides of WPRE (ab-WPRE-ab: 90.3% ± 7.3%, *n* = 5 hemispheres), compared with the previously reported AAV with WPRE-ab (7.9% ± 3.6%, *n* = 5 hemispheres) (∗∗∗∗*p* < 0.0001 by Bonferroni’s multiple comparisons test following one-way ANOVA) (Figure 2D). Notably, ab-WPRE-ab showed significantly higher microglial specificity than WPRE-ab (∗∗∗*p* < 0.001).

Additionally, the efficiency of GFP expression in microglia significantly increased to 61.0% ± 12.1% with ab-WPRE (*n* = 6 hemispheres) and 61.5% ± 12.9% with ab-WPRE-ab (*n* = 5 hemispheres) compared with WPRE-ab (11.1% ± 8.3%, *n* = 5 hemispheres) (∗∗∗∗*p* < 0.0001 vs. WPRE-ab by Bonferroni’s multiple comparisons test following one-way ANOVA) (Figure 2E). There were no significant differences between ab-WPRE and ab-WPRE-ab in terms of microglial transduction efficiency or GFP fluorescence intensity within transduced microglia (*p* > 0.9999 by Bonferroni’s test and *p* = 0.3122 by unpaired t test, respectively) (Figures 2E and 2F).

The expression levels of GFP in microglia were thought to be higher in AAVs with miR.T sequences upstream of WPRE compared with AAVs with miR.T sequences only downstream of WPRE. This is because GFP fluorescence was clearly observable without immunohistochemistry in sections treated with AAVs containing miR.T sequences upstream of WPRE (ab-WPRE and ab-WPRE-ab), whereas it was scarcely detectable in sections treated with AAVs containing miR.T sequences only downstream of WPRE (WPRE-ab) (Figure S2).

We next investigated whether increasing the dose of AAV.mIba1.GFP.ab-WPRE-ab would further enhance transduction efficiency in microglia. A high dose of the vector (5.0E+12 vg/mL, 0.5 μL) was injected into the mouse cerebral cortex, and immunohistochemistry was performed 3 weeks later. Under these conditions, off-target expression in neurons was observed, resulting in decreased specificity (64.7% ± 8.6%, *n* = 9 hemispheres) (Figure S3). Nevertheless, the specificity remained markedly higher than that reported in a previous study using WPRE-ab (∼4%).^15^

The higher microglial specificity of ab-WPRE-ab compared with ab-WPRE is unlikely to be due to the presence of two copies of miR.T, as placing two copies of miR.T upstream of WPRE (abab-WPRE) did not improve neuron detargeting (Figure S4).

### The Iba1 promoter is indispensable for transgene expression in microglia

Given the significant enhancement in microglial specificity with miR.T sequences placed upstream of WPRE, we next investigated the necessity of the mIba1 promoter for microglia-specific gene expression. We replaced the mIba1 promoter with the ubiquitously active cytomegalovirus early enhancer/chicken β-actin (CAG) promoter in AAV vectors expressing GFP-ab-WPRE-ab-poly(A) and injected these into the cerebral cortex of mice.

**Figure 3:**
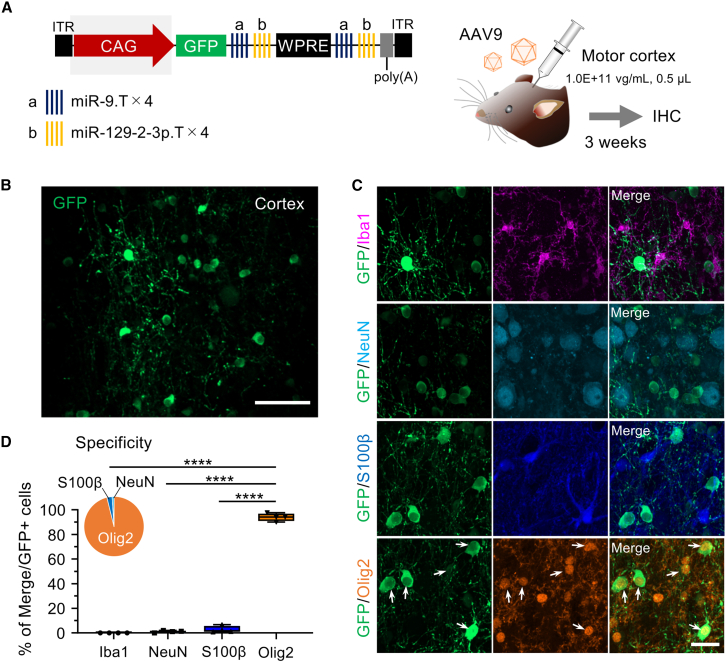
Oligodendrocyte-specific GFP expression by AAV.CAG.GFP.ab-WPRE-ab (A) Schematic depicting the AAV genome comprising microRNA target sequences on both sides of WPRE and expressing GFP under the CAG promoter. Mice received an injection of the AAV9 vectors (1.0E+11 vg/mL, 0.5 μL) into the motor cortex. Mice were euthanized 3 weeks post-injection and analyzed by immunohistochemistry. (B and C) Low-magnification fluorescent images immunolabeled for GFP (B) and enlarged images of the transduced areas immunostained for GFP, Iba1, NeuN, S100β, and Olig2 (C). Arrows indicate Olig2-immunolabeled GFP-expressing oligodendrocytes. Scale bars, 100 μm (B) and 20 μm (C). (D) Box-and-whisker graph and pie chart showing the percentage of each cell type among total GFP-expressing cells (*n* = 4 hemispheres). Note that almost all GFP-expressing cells are Olig2-labeled oligodendrocytes. ∗∗∗∗*p* < 0.0001 by Bonferroni’s multiple comparisons test following one-way ANOVA. The box-and-whisker plots depict the median (centerlines), 25th and 75th percentiles (bounds of the box), and minimum/maximum values (whiskers).

In the initial pilot experiment, administration at the same titer as the AAV with the mIba1 promoter (1.0E+12 vg/mL, 0.5 μL) resulted in cellular damage near the injection site, including morphological alterations of various cell types, and notable GFP expression in surrounding neurons. Therefore, the titer was reduced to one-tenth (1.0E+11 vg/mL, 0.5 μL) for subsequent injections (Figure 3A). Notably, we found that GFP was not expressed in microglia, but instead was predominantly expressed in oligodendrocytes and, to a lesser extent, astrocytes (Figures 3B–3D). These results suggest that the mIba1 promoter is essential for achieving microglia-specific gene expression in combination with miR.T sequences.

### High microglial specificity of AAV with miR.T on both sides of WPRE in the striatum and cerebellum

**Figure 4:**
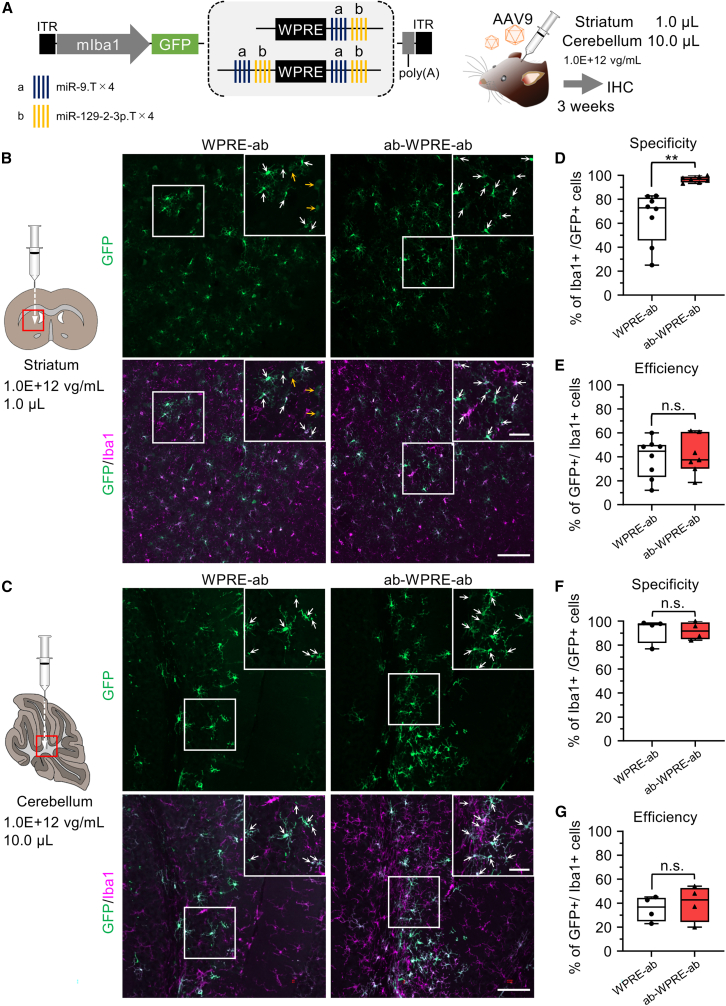
Microglia-specific GFP expression in the striatum and cerebellum by AAV.mIba1.GFP.ab-WPRE-ab (A) AAV9.mIba1 carrying ab-WPRE-ab was injected into the striatum (1.0E+12 vg/mL, 1.0 μL) or cerebellum (1.0E+12 vg/mL, 10 μL) of C57BL/6J mice. Three weeks after the injection, the mice were euthanized and analyzed by immunohistochemistry. (B and C) Confocal microscopy of striatal (B) and cerebellar (C) sections. The left and right panels show sections from mice injected with AAV.mIba1.WPRE-ab (left) and AAV.mIba1.ab-WPRE-ab (right). Boxed areas are expanded in the upper right corner of each panel, where white and yellow arrows indicate GFP-expressing microglia and neurons, respectively. Scale bars, 100 μm (bottom right) and 40 μm (inset). (D–G) Summarized graphs showing the specificity (D and F) and efficiency (E and G) of GFP expression in microglia in the striatum (D and E; *n* = 8 hemispheres for WPRE-ab, *n* = 7 hemispheres for ab-WPRE-ab) and cerebellum (F and G; *n* = 4 hemispheres for WPRE-ab, *n* = 4 hemispheres for ab-WPRE-ab). ∗∗*p* < 0.01; n.s., not significant (*p* > 0.05) by unpaired t test. The box-and-whisker plots depict the median (centerlines), 25th and 75th percentiles (bounds of the box), and minimum/maximum values (whiskers).

To investigate whether the optimized AAV vector (AAV.mIba1.GFP.ab-WPRE-ab) could achieve microglia-specific transgene expression in other brain regions, we injected this vector into the striatum (*n* = 7 hemispheres) and cerebellum (*n* = 4 mice) of mice (Figure 4A). Three weeks post-injection, we observed highly specific GFP expression in microglia in both brain regions (Figures 4B and 4C). Compared with the control AAV.mIba1.GFP.WPRE-ab, the optimized AAV.mIba1.GFP.ab-WPRE-ab exhibited significantly higher specificity for microglia in the striatum (96.7% ± 2.3%, *n* = 7 hemispheres vs. 64.8% ± 20.0%, *n* = 8 hemispheres for control, ∗∗*p* < 0.01 by unpaired t test) and almost comparable specificity for cerebellar microglia (91.9% ± 6.3%, *n* = 4 mice vs. 92.4% ± 9.0%, *n* = 4 mice for control) (Figures 4D and 4F).

The efficiency of GFP expression in microglia was comparable between the two AAV vectors in both the striatum and cerebellum (Str. 38.8% ± 15.4%, *n* = 8 hemispheres; Cbl. 35.4% ± 9.0%, *n* = 4 mice for WPRE-ab and Str. 41.2% ± 14.6%, *n* = 7 hemispheres; Cbl. 39.9% ± 13.0%, *n* = 4 mice for ab-WPRE-ab) (Figures 4E and 4G). Therefore, it is suggested that ab-WPRE-ab is capable of microglia-specific expression in other brain regions. The lack of a significant difference in microglial specificity between WPRE-ab and ab-WPRE-ab in the cerebellum may be because the mIba1 promoter already achieves high specificity without the need for miR.T in the cerebellum.^15^

### Sustained microglia-specific GFP expression 2 months post-injection

**Figure 5:**
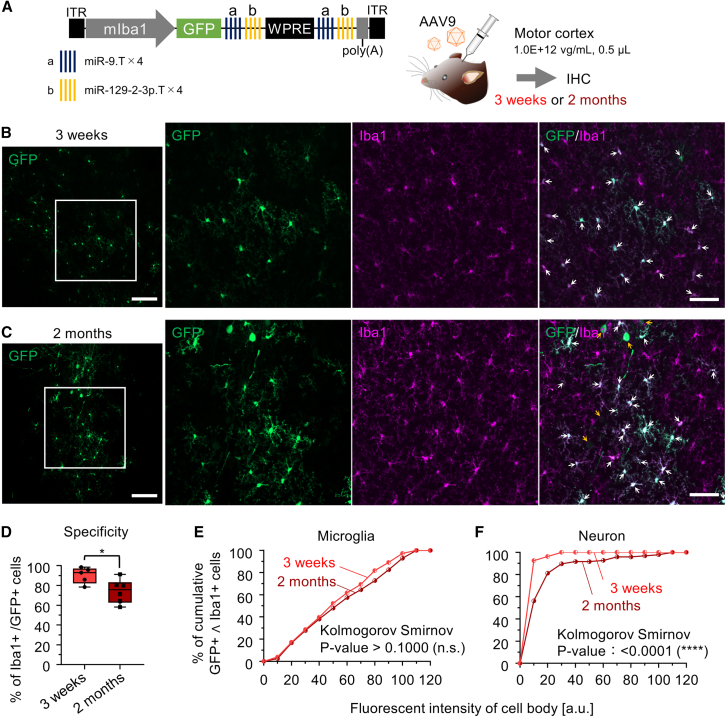
Maintenance of GFP expression specificity in microglia 2 months after AAV injection (A) AAV.mIba1.GFP.ab-WPRE-ab (1.0E+12 vg/mL, 0.5 μL) was injected into the motor cortex of C57BL/6J mice. The mice were euthanized 3 weeks or 2 months after the injection and analyzed by immunohistochemistry. (B and C) Confocal microscopy of cerebral sections 3 weeks (B) and 2 months (C) post-injection. The two panels on the left are low-magnification images, while the panels on the right are magnified images of the boxed areas in the left panels. White and yellow arrows indicate GFP-expressing microglia and non-microglial cells, respectively. Scale bars, 100 μm (left) and 50 μm (right). (D) Graph showing the specificity of GFP expression in microglia 3 weeks (*n* = 5 hemispheres) or 2 months (*n* = 6 hemispheres) after AAV injection. The data for the 3-week time point is the same as in Figure 2D. ∗*p* < 0.05 by unpaired t test. The box-and-whisker plots depict the median (centerlines), 25th and 75th percentiles (bounds of the box), and minimum/maximum values (whiskers). (E and F) Cumulative plot of GFP fluorescence intensity in the cell bodies of GFP- and Iba1-double-positive microglia (E) or in the cell bodies of GFP-positive and Iba1-negative neurons (F). Red and dark red symbols indicate results obtained from mice 3 weeks after injection (285 microglial cell bodies) (E) and (27 neuronal cell bodies) (F) from 5 hemispheres and results obtained from mice 2 months after injection (296 microglial cell bodies) (E) and (96 neuronal cell bodies) (F) from 6 hemispheres, respectively. *p* > 0.1 (n.s., not significant) for (E) and ∗∗∗∗*p* < 0.0001 for (F) by Kolmogorov-Smirnov test.

To evaluate the long-term persistence of microglia-specific gene expression, we injected AAV.mIba1.GFP.ab-WPRE-ab into the cerebral cortex and analyzed the brains 2 months post-injection (*n* = 6 hemispheres) (Figure 5A). Although the frequency of GFP-positive non-microglial cells increased over time, GFP-expressing microglia still accounted for approximately 74.2% ± 11.1% of the total GFP-expressing cells (*n* = 6 hemispheres) (Figures 5B–5D). This suggests that, while some leakage into non-microglial cells occurs over time, the vector retains high specificity for microglia over an extended period.

The intensity of GFP fluorescence in microglia did not significantly change between the 3-week and 2-month time points, indicating that transgene expression levels remained stable in microglia over time (*p* > 0.1 by Kolmogorov-Smirnov test) (Figure 5E). In contrast, GFP fluorescence intensity in neurons increased significantly (∗∗∗∗*p* > 0.0001 by Kolmogorov-Smirnov test), suggesting that the gradual reduction in microglial specificity was due to increased transgene expression in non-target neurons (Figure 5F).

### Application of our optimized microglia-selective gene expression method to physiological experiments in cortical microglia

**Figure 6:**
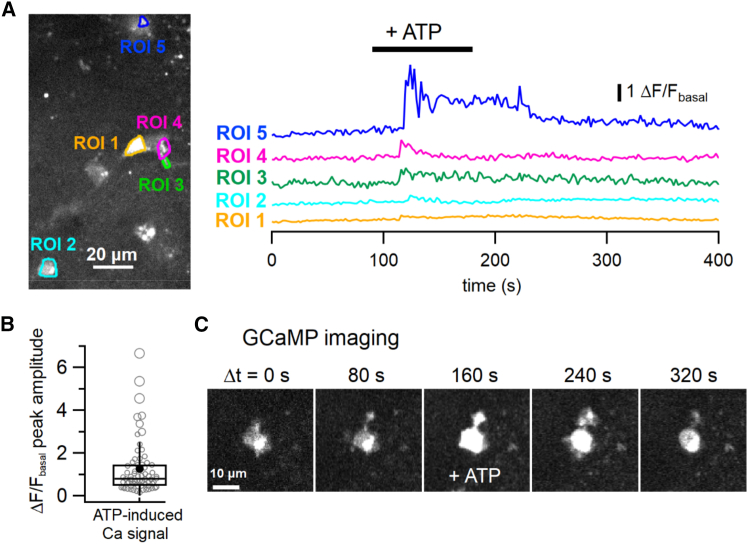
Measurement of Ca^2+^ signals in cortical microglia expressing the genetically encoded Ca^2+^ indicator jGCaMP8s using our microglia-selective AAV-mediated gene expression method (A) The left panel displays an averaged confocal image of virally expressed jGCaMP8s signals in the motor cortex of an acute cerebral slice. Regions of interest (ROIs 1–5) were placed on microglial compartments. The right panel shows Ca^2+^ signal traces estimated from the ROIs in the left panel. Bath application of 100 μM ATP (for 90 s, indicated by the black bar) induced Ca^2+^ transients in the transduced microglia. Scale bar, 20 μm. (B) A box-and-whisker plot showing the peak amplitude of quantified Ca^2+^ signals induced by bath-applied ATP (ΔF/F_basal_; see STAR Methods) in microglial compartments. Open circles represent individual data points, while the horizontal line and the box represent the median value and interquartile range, respectively (*n* = 68 cellular compartments from 8 cerebral slices of 5 mice). The error bars extend one standard deviation above and below the mean (filled circle). (C) Time-lapse GCaMP images (single focal plane) capturing both the movement of microglial processes and ATP-induced Ca^2+^ increases in the microglia. Scale bar, 10 μm. See also Video S1.

Next, we examined whether our optimized microglia-selective gene expression method could be applied to physiological experiments involving microglia in the motor cortex. First, we attempted to measure Ca^2+^ signals through AAV-mediated expression of the genetically encoded fluorescent calcium indicator, jGCaMP8s, in cortical microglia of the primary and secondary motor areas.^22^ Three to 4 weeks after AAV injection into the motor cortex, we performed confocal live Ca^2+^ imaging in acute cerebral slices, where GCaMP-positive microglia were observed using our updated method (Figure 6A).

Bath application of ATP (100 μM) induced a Ca^2+^ increase in cellular compartments, including microglial cell bodies and processes, with a variable delay (Figure 6A; Video S1). This delay is likely due to the time required for solution exchange and the microglial response. The mean peak amplitude of ATP-induced Ca^2+^ signal changes (ΔF/F_basal_; see STAR Methods) was 1.26 ± 0.15 (Figure 6B; *n* = 68 cellular compartments from 8 cerebral slices of 5 mice). These results align with typical microglial Ca^2+^ dynamics, as microglia express purinergic receptors and exhibit ATP-induced Ca^2+^ responses within tens of seconds.^23^ Additionally, some GCaMP-positive cells exhibited process movement and ATP-induced process extension (Figure 6C; Video S1), which are hallmark features of microglia.^24^

Video S1. GCaMP images illustrating microglial process movements and ATP-induced Ca^2+^ response, related to Figure 6 This video shows GCaMP images illustrating microglial process movements and ATP-induced Ca^2+^ response as depicted in Figure 6C. Exogenous ATP (100 μM) was bath applied for 1.5 min, as indicated in the video. The video is played at 30 frames per second (60 times faster than real time). Scale bar, 10 μm.

Since the GCaMP signals in Figure 6C reflect both the movement of the processes and changes in Ca^2+^ concentration within the processes, it is difficult to discern whether the processes themselves are moving or only the Ca^2+^ concentration is changing. To more accurately capture dynamic morphological changes and process movements in microglia, we specifically expressed GFP in cerebral microglia using our method and performed live GFP imaging with confocal microscopy (Figure S5). Most GFP-positive microglia exhibited clear basal morphological motility, although there was some variability among individual cells (Figure S5; Videos S2, S3, S4, and S5). In many cases, it was difficult to reliably capture the complete three-dimensional (3D) movement of a single microglial process within a single focal plane (Figures S5A and S5B, upper panels; Video S6). To overcome this, we acquired z stack images (typically more than 25 images), covering the entire structure of a single microglia at each time point, and created time-lapse 2D images from the maximum intensity projections of the z stacks (Figures S5A and S5B, lower panels).

Video S2. Basal microglial process movements observed in a single focal plane, related to Figure S5A This time-lapse GFP video shows an example of basal microglial process movements within a single focal plane in the motor cortex. The video is played at 60 frames per second (120 times faster than real time). Scale bar, 10 μm.

Video S3. Another example of basal microglial process movements observed in a single focal plane, related to Figure S5A The conditions of the recording and video presentation are similar to Video S2.

Video S4. *z* axis maximum intensity projections captured basal movements of whole microglial processes, related to Figure S5A This 2D time-lapse image of *z* axis maximum intensity projections across multiple focal planes clarifies basal movements of whole microglial processes in the motor cortex. Notably, putative microglial cell bodies near the center remained static in the projected videos. The video is played at 15 frames per second (100 times faster than real time). Scale bar, 10 μm.

Video S5. Another example of *z* axis maximum intensity projections that captured basal movements of whole microglial processes, related to Figure S5A The conditions of the recording are similar to Video S4. The video is played at 15 frames per second (120 times faster than real time). Scale bar, 10 μm.

Video S6. ATP-induced microglial process extension observed by single focal plane imaging, related to Figure S5 This video of GFP images shows ATP-induced microglial process extension (single focal plane) as depicted in Figure S5B (upper panel). The extended microglial processes reached the focal plane and became visible with a slight delay following bath application of ATP. The video is played at 60 frames per second (120 times faster than real time). Scale bar, 10 μm.

Time-lapse 2D images from multiple focal planes showed that microglial cell bodies remained relatively static, while the processes exhibited dynamic motility (Figures S5A and S5B, arrowheads; Videos S4, S5, S7, andS9). Bath application of ATP (100 μM) induced pronounced elongation of microglial processes after a delay (Figure S5B; Videos S6, S7, S8, and S9), consistent with the typical morphological dynamics of microglia.^24^^,^^25^ These results suggest that our optimized microglia-selective expression method is effective for live imaging experiments investigating the morphological dynamics of microglia. Taken together, we conclude that our updated AAV-mediated, microglia-specific gene expression method is also applicable to physiological experiments.

Video S7. *z* axis maximum intensity projections reveal ATP-induced extension and subsequent increased motility of microglial processes in the motor cortex, related to Figure S5B This video is composed of 2D time-lapse GFP images constructed from the maximum intensity projections of z stacks acquired from putative single microglia. The video demonstrates ATP-induced extension and subsequent increased motility of microglial processes in the motor cortex. The video is played at 30 frames per second (180 times faster than real time). Scale bar, 10 μm.

Video S8. Another example of *z* axis maximum intensity projections that reveal ATP-induced extension and subsequent increased motility of microglial processes in the motor cortex, related to S5B The conditions of the recording and video presentation are similar to Video S7.

Video S9. Another example of *z* axis maximum intensity projections that reveal ATP-induced extension and subsequent increased motility of microglial processes in the motor cortex, related to Figure S5B The conditions of the recording and video presentation are similar to Video S7.

### Successful microglial transgene expression by intravenous injection of AAV.mIba1 harboring ab-WPRE-ab

We next investigated whether our updated AAV vector could achieve microglia-specific gene expression through intravenous injection. To this end, we used five different BBB-penetrating capsid variants—PHP.B,^26^ PHP.eB,^27^ 9P31,^28^ and two others that were reported as BBB-penetrating and microglia targeting capsids (AAV9-HGTAASH/YAFGGEG (AAV(H)/AAV(Y)))—to package the AAV genome (Figures S6A and S6B).^17^ AAV(H) and AAV(Y) capsid variants feature a seven-amino-acid insertion, (HGTAASH) or (YAFGGEG), between amino acids 588 and 589 of the AAV9 capsid. These variants have been shown to deliver transgenes to microglia with up to 80% efficiency following intravenous injection.^17^ Mice received intravenous injection of one of these AAV vectors (2.0E+13 vg/mL, 100 μL). Three weeks post-injection, brain sections were analyzed by immunohistochemistry.

**Figure 7:**
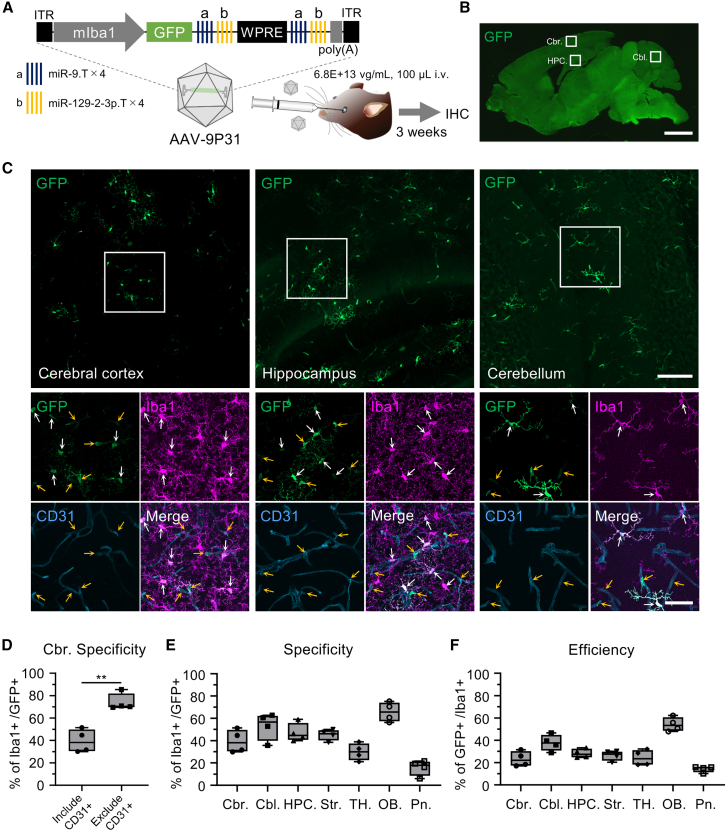
GFP expression in microglia throughout the brain following intravenous injection of AAV-9P31.mIba1.GFP.ab-WPRE-ab (A) The microglia-targeting AAV genome containing mIba1.GFP.ab-WPRE-ab was packaged with the mouse BBB-permeable AAV-9P31 capsid. The AAV vector (6.8E+13 vg/mL, 100 μL) was injected intravenously into C57BL/6J mice via the orbital venous plexus. The mice were euthanized 3 weeks after injection and analyzed by immunohistochemistry. (B) Sagittal section of the whole brain immunolabeled for GFP. Scale bar, 2 mm. (C) The top three images, immunolabeled for GFP, correspond to the boxed areas of the cerebral cortex, hippocampus, and cerebellum in the sagittal section (B). The bottom four images, immunolabeled for GFP, Iba1, and CD31, are magnifications of the boxed areas in the top GFP-labeled images. White and yellow arrows indicate GFP-expressing microglia and non-microglial cells, respectively. Note that most GFP-positive non-microglial cells are CD31-positive vascular endothelial cells. Scale bars, 100 μm (upper right) and 40 μm (lower right). (D) Graph depicting the specificity of GFP expression in cortical microglia. “Include CD31+” same as data in (E) Cbr. and “Exclude CD31+” represent the microglial specificity of GFP expression when calculated including CD31-positive endothelial cells (*n* = 4 mice) and excluding CD31-positive endothelial cells (*n* = 4 mice), respectively. ∗∗*p* < 0.01 by unpaired t test. (E and F) Specificity (E) and efficiency (F) of GFP expression in microglia across various brain regions following intravenous injection of the microglia-targeting AAV-9P31 vectors (n = 4 mice). Cbr., cerebrum; Cbl., cerebellum; HPC., hippocampus; Str., striatum; TH., thalamus; OB., olfactory bulb; Pn., pons. See also Figures S6–S8. (D–F) The box-and-whisker plots depict the median (centerlines), 25th and 75th percentiles (bounds of the box), and minimum/maximum values (whiskers).

Fluorescence microscopy revealed that brain sections from mice injected with PHP.B, PHP.eB, and AAV(H)/AAV(Y) vectors had few GFP-labeled cells, whereas sections from mice injected with the AAV-9P31 vector showed more GFP-expressing microglia, although the signal was faint (Figures S6C–S6G). To enhance transgene expression and more clearly label microglia, we increased the injection dose of the AAV-9P31 vector to 6.8E+13 vg/mL (100 μL) and repeated the experiment (Figure 7A).

Confocal microscopy of brain sections from mice that received higher doses of AAV-9P31 vectors showed numerous GFP-labeled cells throughout the brain (Figures 7B, 7C, and S7B–S7F). Immunohistochemical analysis revealed that the GFP-expressing cells were primarily Iba1-positive microglia and CD31-positive vascular endothelial cells. Quantification of the immunohistochemistry showed that the specificity of GFP expression for microglia in the cerebral cortex was approximately 40% (39.4% ± 8.9%, *n* = 4 mice). When the analysis was limited to the brain parenchyma, excluding vascular endothelial cells, the specificity of GFP expression for microglia in the cerebral cortex increased to 74% (74.0% ± 6.6%, *n* = 4 mice, ∗∗*p* < 0.01 by unpaired t test) (Figure 7D). The specificity and efficiency of transgene expression for microglia across different brain regions are summarized in Figures 7E and 7F, with the olfactory bulb exhibiting the highest specificity and efficiency among the regions examined (specificity: Cbr. 39.4% ± 8.9%, Cbl. 53.0% ± 10.6%, HPC. 47.1% ± 7.3%, Str. 45.3% ± 4.2%, TH. 30.0% ± 6.6%, OB. 65.7% ± 7.5%, Pn. 15.9% ± 6.0% [*n* = 4 mice]; efficiency: Cbr. 23.3% ± 5.9%, Cbl. 37.8% ± 6.4%, HPC. 28.2% ± 3.5%, Str. 26.4% ± 3.9%, TH. 24.4% ± 6.1%, OB. 54.2% ± 5.4%, Pn. 14.4% ± 2.4% [*n* = 4 mice]).

### AAV9-9P31 works in middle-aged microglia

To assess the efficacy of the AAV9-9P31 vector in aged animals, we intravenously administered the construct (6.8E+13 vg/mL, 100 μL) to a 36-week-old mouse and examine GFP expression profile by immunohistochemistry. Although the overall transduction efficiency was lower than in younger mice, specific GFP expression in microglia was still readily detectable. Notably, we also observed a marked reduction in GFP expression in vascular endothelial cells in aged animals (Figure S8).

## Discussion

In this study, we demonstrated that relocating miR-9.T and miR-129.T from the 3′ to the 5′ side of WPRE significantly enhances both the specificity and efficiency of transgene expression in cortical microglia. Furthermore, placing miR.T sequences on both the 5′ and 3′ sides of WPRE further improved microglial specificity, which remained robust even 2 months post-AAV injection. Since miRs bind to and cleave their perfectly complementary sequences,^19^^,^^20^ the miR.T sequences located downstream of WPRE are cleaved, producing GFP mRNA with WPRE, whereas cleavage of upstream miR.T sequences results in GFP mRNA alone (Figures S1B and S1C). WPRE stabilizes the mRNA,^21^ preventing degradation and facilitating protein translation. In contrast, GFP mRNA lacking both WPRE and poly(A) sequences is rapidly degraded, leading to no GFP protein expression.

Transgene specificity for microglia was notably higher in AAV constructs with miR.T on both sides of WPRE compared with constructs with miR.T only on the 5′ side (Figure 2D). This increase is likely because the miR.T on the 3′ side acts as a safeguard for the 5′ miR.T. Without the 3′ miR.T, incomplete cleavage at the 5′ miR.T produces mRNA containing GFP, WPRE, and poly(A), resulting in strong GFP protein expression. However, the presence of the 3′ miR.T ensures that any uncleaved mRNA at the 5′ miR.T is cleaved at the 3′ miR.T, producing mRNA consisting only of GFP and WPRE sequences, which significantly suppresses GFP protein expression due to the absence of the poly(A) signal.

In addition to increasing specificity, placing the miR.T sequences upstream of WPRE also unexpectedly enhanced the efficiency of transgene expression in microglia (Figure 2E). One possible explanation is that the insertion of miR.T sequence between the coding sequence and WPRE introduces spacing that stabilizes the viral mRNA structure in microglia, where these miRs are not active. This configuration may reduce the formation of inhibitory RNA secondary structures or shield WPRE from structural interference, resulting in improved mRNA stability and translation efficiency. In contrast, in neurons, the viral mRNA is efficiently cleaved and degraded due to active miR-9 and miR-129-2-3p, minimizing off-target expression. This structural hypothesis might explain the dual effect of enhanced specificity and expression efficiency observed with the upstream placement of miR target sites.

A major limitation of the current system is the cargo capacity imposed by the AAV packaging limit. Considering the required regulatory elements—including ITRs, the mIba1 promoter, miR.T sequences, WPRE, and poly(A)—the available space for the transgene is approximately 1.8 kb. Even the smallest genome editors, such as cjCas9 (2,949 bp), exceed this limit and cannot be directly included in the current cassette.

One possible strategy to secure additional space would be to omit WPRE, retaining only the miR.T sequences and a polyadenylation signal downstream of the transgene. This modification could save ∼600 bp, enabling the inclusion of larger payloads. However, WPRE has been shown to play a critical role in enhancing transgene expression. For example, in cultured hippocampal neurons (albeit using a different promoter), removal of WPRE reduced GFP expression to ∼20%–30% of that seen with WPRE.^29^ Our unpublished data using a CaMKII promoter-driven AAV-PHP.B vector further support this, showing a marked decrease in GFP expression in the brain upon WPRE deletion following intravenous administration. Therefore, while omitting WPRE might theoretically allow larger payloads, we consider it indispensable for achieving robust transgene expression in microglia.

To enable genome editing within these spatial constraints, future directions could include promoter miniaturization or dual-vector systems. For instance, an mIba1-driven tTA AAV paired with a TRE-controlled genome editor AAV may offer a feasible solution, although such strategies would require careful balancing of co-transduction efficiency and packaging space.

A related concern is whether microglial gene expression profiles are altered as a result of AAV transduction. Indeed, it is possible that AAV capsid proteins or single-stranded viral DNA may activate innate immune signaling pathways in microglia, leading to transcriptional changes. However, this is not a phenomenon limited to our microglia-targeting construct; multiple studies have shown that commonly used AAV serotypes, including AAV9, also transduce microglia to some extent, and may elicit similar responses. Additionally, direct intracerebral AAV injection can cause transient tissue damage and inflammation, leading to morphological activation of microglia accompanied by a ramified-to-amoeboid morphological transformation. Importantly, a previous study has demonstrated that these inflammatory effects subside within 3 weeks, with both microglial morphology and density returning to baseline.^30^ Based on these findings, we recommend evaluating transgene expression at least 3 weeks after the administration of our microglia-targeting AAV vectors in order to minimize confounding effects from innate immune activation or local tissue responses. This timing helps ensure that observed expression reflects stable gene delivery rather than transient immune or inflammatory effects.

Although the CAG promoter is widely considered to be a ubiquitous and robust promoter, our AAV-based experiments, which replaced the mIba1 promoter in the microglia-targeting cassette with the CAG promoter (Figure 3), showed clear expression in oligodendrocytes but not in microglia. This contrasts with its well-documented activity in transgenic reporter lines such as Ai14, which has been used in combination with P2ry12-CreER or Cx3cr1-CreER to effectively label microglia.^31^^,^^32^ One possible explanation for this discrepancy lies in the difference between chromosomally integrated vs. episomal (AAV-based) transgene expression systems. In episomal contexts, the local chromatin environment, DNA accessibility, or epigenetic silencing in microglia may hinder CAG promoter activity, despite its broad utility in other systems.^33^ Indeed, prior studies have reported that promoter activity can vary across cell types depending on both the vector type and delivery method: AAV1-CAG promoter-GFP exhibited efficient GFP expression in mouse neurons, astrocytes, and oligodendrocytes, but no expression was observed in microglia.^34^ Thus, our findings may not contradict those using transgenic lines but rather reflect inherent biological differences between vector systems.

Previously, we reported that intravenous injection of AAV-PHP.B.mIba1.GFP.WPRE-ab resulted in residual GFP aggregates in microglial lysosomes.^15^ In this study, we utilized five different BBB-penetrating capsid variants to investigate whether intravenous administration of a GFP-expressing AAV with miR.T sequences on both sides of WPRE could specifically and efficiently label microglia. Our results demonstrated that AAV-9P31, AAV-PHP.B, and AAV-PHP.eB expressed GFP in the cytoplasm of microglia, with AAV-9P31 showing the highest efficiency. Further investigation using high doses of AAV-9P31.mIba1.GFP.ab-WPRE-ab revealed microglial specificity ranging from 20% in the pons to 70% in the olfactory bulb, with transduction efficiency ranging from 20% (pons) to 60% (olfactory bulb).

One potential explanation for the inconsistency between our findings and previously reported microglia-targeting AAV variants may lie in differences in vector production methods.^17^ In our protocol, we culture HEK293 cells in serum-free conditions after transfection and harvest AAV particles exclusively from the culture supernatant. In contrast, some previously reported preparations may involve capsid isolation from cell lysates. This difference in harvesting strategy could lead to variations in capsid-associated post-translational modifications, such as glycosylation, which may influence BBB permeability and cell-type tropism. Supporting this notion, we observed that a BBB-penetrant AAV vector (not an AAV(H)/AAV(Y)), which was provided by another laboratory and purified from cell lysates, exhibited a moderately but significantly higher brain transduction efficiency following intravenous administration, compared with our supernatant-purified preparation (data not shown). These findings underscore the importance of vector production methods when evaluating AAV tropism and should be taken into consideration when interpreting reproducibility across studies.

Notably, intravenous administration of AAV-9P31.mIba1.GFP.ab-WPRE-ab resulted in highly efficient GFP expression in vascular endothelial cells across all brain regions examined. These findings suggest that the mIba1 promoter is active in vascular endothelial cells, where miR-9 and miR-129-2-3p are not endogenously expressed, leading to unregulated transgene expression. When transgene expression in vascular endothelial cells was excluded, GFP expression specificity in cortical microglia reached nearly 75% (Figure 7D), suggesting that intravenous administration of AAV-9P31.mIba1.ab-WPRE-ab enables microglia-selective transgene expression in the brain parenchyma. Incorporating sequences complementary to miRs expressed in vascular endothelial cells but not in microglia into the AAV vector may help suppress transgene expression in endothelial cells and further enhance microglial specificity.

Here, by placing miR.T on both sides of WPRE in AAV vectors, we significantly improved both the specificity and efficiency of transgene expression in cortical microglia over an extended period. These enhanced microglia-targeting AAV vectors will be valuable tools for studying microglial physiology and pathophysiology, as well as for developing microglia-targeted gene therapies for various neuropsychiatric diseases involving microglia.

### Limitations of the study

While our optimized AAV vector enables highly specific and efficient gene expression in microglia *in vivo*, several limitations remain. First, the total packaging capacity of the AAV genome (∼4.7 kb) constrains the size of transgenes that can be delivered (∼1.8 kb), as the mIba1 promoter has relatively large size (1.7 kb). Second, while we observed minimal off-target expression in neurons at moderate doses, higher viral titers led to reduced specificity, indicating the need for careful dose optimization. Third, although systemic delivery using a BBB-penetrant capsid enables broad microglial targeting, it results in relatively low efficiency and unintended transgene expression in vascular endothelial cells. Lastly, potential effects of AAV transduction on microglial gene expression and immune activation remain to be fully characterized.

## Resource availability

### Lead contact

Requests for further information and resources should be directed to and will be fulfilled by the lead contact, Hirokazu Hirai (hirai@gunma-u.ac.jp).

### Materials availability

Detailed information on pAAV/mIba1.GFP.miR-9.T.miR-129-2-3p.T.WPRE.miR-9.T.miR-129-2-3p.T.SV40pA, including its sequence, can be obtained from Addgene (plasmid no. 226475). The other plasmids generated in this study will be made available on request, but we may require a payment and/or a completed materials transfer agreement if there is potential for commercial application. There are restrictions to the availability of the AAV vectors because of the lack of an external centralized repository for its distribution and our need to maintain the stock. We are glad to share the vectors with reasonable compensation by requestor for its processing and shipping.

### Data and code availability

• All data reported in this paper will be shared by the lead contact upon request.
• This paper does not report original code.
• Any additional information required to reanalyze the data reported in this work paper is available from the lead contact upon request.

## Acknowledgments

This work was supported by grants from the Program for Brain Mapping by Integrated Neurotechnologies for Disease Studies (Brain/MINDS; JP20dm0207057/JP21dm0207111 to H.H.) and Multidisciplinary Frontier Brain and Neuroscience Discoveries (Brain/MINDS 2.0; JP24wm0625103 to H.H.), from the 10.13039/100009619Japan Agency for Medical Research and Development (10.13039/100009619AMED) and 10.13039/501100001700MEXT/10.13039/501100001691JSPS 10.13039/501100001691KAKENHI (20K06906/24K10022 to N.H., 22K06454/24H01221 to A.K., and 23H02791 to H.H.). The authors thank Asako Ohnishi, Nobue McCullough, and Chieko Miyazawa for AAV vector production; Junko Sugiyama for mouse care; Shota Togai for conducting the preliminary CAG experiments; and Yasunori Matsuzaki and Yuki Fukai for their technical advice on plasmid editing and animal experiments. The graphical abstract includes illustrations created with BioRender.com.

## Author contributions

R.A., A.K., and H.H. designed the experiments. R.A., N.H., and H.K. conducted the research. N.H. performed the Ca^2+^ imaging and live microglia imaging experiments. H.H. supervised and completed the study.

## Declaration of interests

Gunma University, with H.H., A.K., and R.A. as inventors, has filed a patent application in Japan for the microglia-optimized AAV vectors described in this paper (patent application no. 2024-013394).

## STAR★Methods

### Key resources table

**Table:** 

REAGENT or RESOURCE	SOURCE	IDENTIFIER
**Antibodies**		

Anti-GFP	Nacalai Tesque	Cat# 04404-84; RRID:AB_10013361 (See Table S1)
Anti Iba1, Rabbit (for Immunocytochemistry)	FUJIFILM Wako Pure Chemical Corporation	Cat# 019-19741; RRID:AB_839504 (See Table S1)
Anti-NeuN	Millipore	Cat# MAB377; RRID:AB_2298772 (See Table S1)
S100beta antibody	Nittobo Medical	Cat# S100b-Rb-Af1000; RRID:AB_2725784 (See Table S1)
Anti-Olig2 Antibody, clone 211F1.1	Sigma-Aldrich	Cat# MABN50; RRID:AB_10807410 (See Table S1)
GFP (green fluorescent protein) antibody	Nittobo Medical	Cat# GFP-Go-Af1480; RRID:AB_2571574 (See Table S1)
BD Pharmingen™ Purified Rat Anti-Mouse CD31 Clone MEC 13.3	BD Biosciences	Cat# 550274; RRID:AB_393571 (See Table S1)
Donkey anti-Rat IgG (H + L) Highly Cross-Adsorbed Secondary Antibody, Alexa Fluor™ Plus 488	Thermo Fisher Scientific	Cat# A48269; RRID:AB_2893137 (See Table S1)
Donkey anti-Rat IgG (H + L) Highly Cross-Adsorbed Secondary Antibody, Alexa Fluor™ Plus 647	Thermo Fisher Scientific	Cat# A48272; RRID:AB_2893138 (See Table S1)
Donkey anti-Rabbit IgG (H + L) Highly Cross-Adsorbed Secondary Antibody, Alexa Fluor™ Plus 555	Thermo Fisher Scientific	Cat# A32794; RRID:AB_2762834 (See Table S1)
Donkey anti-Mouse IgG (H + L) Highly Cross-Adsorbed Secondary Antibody, Alexa Fluor™ Plus 647	Thermo Fisher Scientific	Cat# A32787; RRID:AB_2762830 (See Table S1)
Donkey anti-Goat IgG (H + L) Highly Cross-Adsorbed Secondary Antibody, Alexa Fluor™ Plus 488	Thermo Fisher Scientific	Cat# A32814; RRID:AB_2762838 (See Table S1)

**Bacterial and virus strains**		

AAV9.mIba1.GFP.WPRE.miR-9.T.miR-129-2-3p.T.SV40pA	Okada et al.^15^	N/A
AAV9.mIba1.GFP.WPRE.miR-708.T.miR-9.T.miR-129-2-3p.T.SV40pA	This paper	N/A
AAV9.mIba1.GFP.WPRE.SV40pA	This paper	N/A
AAV9.mIba1.GFP.WPRE	This paper	N/A
AAV9.mIba1.GFP	This paper	N/A
AAV9.mIba1.GFP.miR-9.T.miR-129-2-3p.T.WPRE.SV40pA	This paper	N/A
AAV9.mIba1.GFP.miR-9.T.miR-129-2-3p.T.WPRE.miR-9.T.miR-129-2-3p.T.SV40pA	This paper	N/A
AAV9.mIba1.GFP.miR-9.T.miR-129-2-3p.T. miR-9.T.miR-129-2-3p.T.WPRE.SV40pA	This paper	N/A
AAV9.CAG.GFP.miR-9.T.miR-129-2-3p.T.WPRE.miR-9.T.miR-129-2-3p.T.SV40pA	This paper	N/A
AAV9.mIba1.jGCaMP8s.WPRE.miR-miR-9.T.miR-129-2-3p.T.SV40pA	This paper	N/A
AAV-9P31.mIba1.GFP.miR-9.T.miR-129-2-3p.T.WPRE.miR-9.T.miR-129-2-3p.T.SV40pA	This paper	N/A
PHP.B.mIba1.GFP.miR-9.T.miR-129-2-3p.T.WPRE.miR-9.T.miR-129-2-3p.T.SV40pA	This paper	N/A
PHP.eB.mIba1.GFP.miR-9.T.miR-129-2-3p.T.WPRE.miR-9.T.miR-129-2-3p.T.SV40pA	This paper	N/A
AAV(H).mIba1.GFP.miR-9.T.miR-129-2-3p.T.WPRE.miR-9.T.miR-129-2-3p.T.SV40pA	This paper	N/A
AAV(Y).mIba1.GFP.miR-9.T.miR-129-2-3p.T.WPRE.miR-9.T.miR-129-2-3p.T.SV40pA	This paper	N/A

**Chemicals, peptides, and recombinant proteins**		

Dulbecco’s phosphate-buffered saline (D-PBS(−))	Fujifilm Wako Pure Chemical Corporation	Cat# 045-29795
Polyethylenimine (PEI) "Max"	Polysciences Inc.	Cat# 24765-1
Dulbecco’s Modified Eagle’s Medium (DMEM)	Sigma-Aldrich	Cat# D5796-500ML
Fetal Bovine Serum	Cosmo Bio	Cat# CCP-FBS-BR-500
Polythelene Glycol 8000	Sigma-Aldrich	Cat# P5413
Iodixanol (Optiprep)	Alere Technologies	Cat# AXS-1114542-250ML

**Critical commercial assays**		

In-Fusion HD Cloning Kit	Takara Bio	Cat# 639649
Power SYBR Green Master Mix	Thermo Fisher	Cat# 4367659

**Experimental models: Cell lines**		

HEK293T Cells	Thermo Fisher Scientific	Cat# HCL4517

**Experimental models: Organisms/strains**		

Mouse: C57BL/6J	Mice bred in our laboratory after purchased from Jackson Laboratory	N/A

**Oligonucleotides**		

Primers targeting the WPRE sequence (Forward; 5′-CTGTTGGGCACTGACAATTC-3′)	This paper	N/A
Primers targeting the WPRE sequence (Reverse; 5′-GAAGGGACGTAGCAGAAGGA-3′)	This paper	N/A
primers targeting GFP (Forward; 5′-CGACCACTACCAGCAGAACAC-3′	This paper	N/A
primers targeting GFP (Reverse; 5′-TGTGATCGCGCTTCTCGTTGG-3′	This paper	N/A

**Recombinant DNA**		

pAAV/mIba1.GFP.WPRE.miR-9.T.miR-129-2-3p.T.SV40pA	Okada et al.^15^	RRID:Addgene_190163
CAG-eYFP-3x-miR708-5p-TS	Challis et al.^18^	RRID:Addgene_117381
pAAV/mIba1.GFP.WPRE.miR-708.T.miR-9.T.miR-129-2-3p.T.SV40pA	N/A	N/A
pAAV/mIba1.GFP.WPRE.SV40pA	Okada et al.^15^	N/A
pAAV/mIba1.GFP.WPRE	This paper	N/A
pAAV/mIba1.GFP.	This paper	N/A
pAAV/mIba1.GFP.miR-9.T.miR-129-2-3p.T.WPRE.SV40pA	This paper	N/A
pAAV/mIba1.GFP.miR-9.T.miR-129-2-3p.T.WPRE.miR-9.T.miR-129-2-3p.T.SV40pA	This paper	RRID:Addgene_226475
pAAV/mIba1.GFP.miR-9.T.miR-129-2-3p.T. miR-9.T.miR-129-2-3p.T.WPRE.SV40pA	This paper	N/A
pAAV/CAG.GFP.miR-9.T.miR-129-2-3p.T.WPRE.miR-9.T.miR-129-2-3p.T.SV40pA	This paper	N/A
pGP-AAV-syn-FLEX-jGCaMP8s-WPRE	Zhang et al.^22^	RRID:Addgene_162377
pAAV/mIba1.jGCaMP8s.miR-9.T.miR-129-2-3p.T.WPRE.miR-9.T.miR-129-2-3p.T.SV40pA	This paper	N/A
pAAV2/9	James Wilson	N/A
AAV-9P31	This paper (sequence based on [Nonnenmacher et al.; patent No. WO2020072683])^28^	N/A
pAAV-PHP.B	Constructed in our laboratory based on the sequence (Deverman et al.^26^; GenBank: KU056473), see (Nitta et al.) for details of the construction process.^35^	N/A
PHP.eB	Constructed in our laboratory based on the sequence (Chan et al.),^27^ see (Konno et al.) for details of the construction process.^36^	N/A
Innate-MGs (AAV(H) and AAV(Y))	This paper (sequence based on [Young et al. ; patent No. WO2022023773])^17^	N/A
pAAV pHelper	Agilent Technologies	Cat# 240071

**Software and algorithms**		

ZEISS ZEN software (ZEN 3.6)	Carl Zeiss	https://portal.zeiss.com
Fiji	ImageJ	https://imagej.net/software/fiji/downloads
Andor iQ3	Andor	https://andor.oxinst.com
Igor Pro9	WaveMetrics	https://www.wavemetrics.com/
Neuromatic	Neuromatic (Rothman et al.)^17^	http://www.neuromatic.thinkrandom.com
Prism (version 9)	GraphPad	https://www.graphpad.com/features
Image Stabilizer plugin	Kang Li, Steven Kang	http://www.cs.cmu.edu/∼kangli/code/Image_Stabilizer.html
Template Matching and Slice Alignment plugin	Qingzong TSENG	https://sites.google.com/site/qingzongtseng/template-matching-ij-plugin#downloads

**Other**		

Vivaspin Turbo 15 MWCO 100000 PES	Takara Bio	Cat# VS15T42

### Experimental model and study participant details

#### Animals

Wild-type C57BL/6J mice which bred in our laboratory after purchased from Jackson Laboratory were used in this study. 6–8 weeks old mice were used in most of the experiments. Only experiments with intravenous injections into aged mouse used 36 weeks female mouse that had finished reproducing. Careful attention was given to the sex of the mice to avoid bias. Mice lived at a room temperature of 23 ± 2°C, 50% humidity, and a light/dark cycle every 12 h. All procedures were performed according to protocols approved by the Japanese Act on the Welfare and Management of Animals and the Guidelines for Proper Conduct of Animal Experiments issued by the Science Council of Japan. The experimental protocol was approved by the Institutional Committee of Gunma University (Nos. 21–065 and 23–057). All efforts were made to minimize suffering and reduce the number of animals used.

#### Cells

HEK293T cells (HCL4517; Thermo Fisher Scientific, Waltham, MA, USA) were cultured in Dulbecco’s Modified Eagle’s Medium (DMEM; D5796-500ML, Merck, Darmstadt, Germany) supplemented with 8% fetal bovine serum (26140-079, Sigma-Aldrich) at 37°C in a humidified atmosphere containing 5% CO_2_. Cells were passaged every 2–3 days upon reaching 70–80% confluency, and maintained for at least 1 week before being used for AAV vector production.

### Method details

#### Vector construction

The plasmid WPRE-ab (pAAV/mIba1.GFP.WPRE.miR-9.T.miR-129-2-3p.T.SV40pA) was obtained from the previous study in our laboratory (Addgene plasmid #190163). The plasmid CAG-eYFP-3x-miR708-5p-TS was a gift from Viviana Gradinaru (Addgene plasmid #117381; http://n2t.net/addgene:117381; RRID:Addgene_117381). The plasmid pGP-AAV-syn-FLEX-jGCaMP8s-WPRE was a gift from the GENIE Project (Addgene plasmid #162377; http://n2t.net/addgene:162377; RRID:Addgene_162377).

To create the plasmid WPRE-cab (pAAV/mIba1.GFP.WPRE.miR-708-5p.T.miR-9.T.miR-129-2-3p.T.SV40pA), the miR-708-5p.T from CAG-eYFP-3x-miR708-5p-TS was amplified by PCR and inserted into WPRE-ab at the KpnI restriction enzyme site using the In-Fusion HD Cloning Kit (639649; Takara Bio, Shiga, Japan). To construct the plasmids ab-WPRE or ab-WPRE-ab (pAAV/mIba1.GFP.miR-9.T.miR-129-2-3p.T.WPRE.SV40pA or pAAV/mIba1.GFP.miR-9.T.miR-129-2-3p.T.WPRE.miR-9.T.miR-129-2-3p.T.SV40pA [Addgene plasmid #226475]), the miR.Ts were placed either upstream or on both sides of the WPRE in pAAV/mIba1.GFP.WPRE.SV40pA. To create the plasmid pAAV/mIba1.jGCaMP8s.miR-9.T.miR-129-2-3p.T.WPRE.miR-9.T.miR-129-2-3p.T.SV40pA, the jGCaMP8s gene was amplified by PCR using pGP-AAV-syn-FLEX-jGCaMP8s-WPRE as a template and inserted into ab-WPRE-ab at the AgeI and NotI restriction enzyme sites. The plasmids pAAV/mIba1.GFP.WPRE and pAAV/mIba1.GFP were created by removing the SV40pA or WPRE-SV40pA, respectively, from pAAV/mIba1.GFP.WPRE.SV40pA.

The Rep/Cap plasmid for pAAV2/9 was kindly provided by Dr. James Wilson. To produce Rep/Cap plasmids for AAV-9P31 or AAV(H)/AAV(Y) (Figure S5), codon-optimized peptide sequences were inserted into the variable region VIII of AAV9. The peptide sequences for AAV(H)/AAV(Y) were obtained from the patent information in WO 2022/023773 A1. The Rep/Cap plasmid for PHP.B was constructed by replacing the pAAV2/9 according to the PHP.B VP1 sequence (GenBank: KU056473) in the previous study.^26^^,^^35^ The Rep/Cap plasmid for AAV-PHP.eB was constructed by replacing the pAAV-PHP.B in the previous study.^27^^,^^36^

#### AAV vector production and titration

Recombinant single-strand AAV vectors were produced using the ultracentrifugation method as described previously.^36^ In brief, three plasmids—the expression plasmid pAAV, pHelper (240071; Agilent Technologies, Santa Clara, CA, USA), and the Rep/Cap plasmid—were co-transfected into HEK293T cells (HCL4517; Thermo Fisher Scientific, Waltham, MA, USA) using polyethylenimine “Max” (24765-1; Polysciences Inc., Warrington, PA, USA). Viral particles were harvested from the culture medium 6 days after transfection and concentrated by precipitation with 8% polyethylene glycol 8000 (P5413; Sigma-Aldrich) and 500 mM sodium chloride. The precipitated AAV particles were resuspended in Dulbecco’s phosphate-buffered saline (D-PBS) and purified with iodixanol (Optiprep; AXS-1114542-250ML; Alere Technologies, Oslo, Norway) using linear density gradient ultracentrifugation with an ultracentrifuge (CP80WX; Himac, Tokyo, Japan). The viral solution was further concentrated and formulated in D-PBS using a Vivaspin Turbo 15 MWCO 100000 PES (VS15T42; Sartorius, Göttingen, Germany).

The genomic titers of the purified AAV vectors, except for AAV9.mIba1.GFP, were determined by quantitative real-time PCR (TP900 or TP970; TaKaRa Bio) using Power SYBR Green Master Mix (Thermo Fisher) with primers targeting the WPRE sequence (5′-CTGTTGGGCACTGACAATTC-3′ and 5′-GAAGGGACGTAGCAGAAGGA-3′). For AAV9.mIba1.GFP, which lacks the WPRE sequence, primers targeting GFP (5′-CGACCACTACCAGCAGAACAC-3′ and 5′-TGTGATCGCGCTTCTCGTTGG-3′) were used.

#### Stereotaxic injection

Mice were anesthetized with intraperitoneal ketamine (80 mg/kg body weight [BW]) and xylazine (8.0 mg/kg BW) and maintained under 0.5% isoflurane anesthesia using an anesthetic vaporizer (MK-AT210D; Muromachi Kikai, Fukuoka, Japan). Anesthetic depth was monitored via the toe-pinch reflex. A hole was created over the injection site using a 30G needle. AAV vectors were injected into the bilateral cerebral cortex (M1-M2) and striatum, or into the cerebellar vermis. A 10-μL Hamilton syringe with a 33G needle was used with a stereotaxic micromanipulator (SMM-100; Narishige, Tokyo, Japan) mounted on a stereotactic frame (SRS-5-HT; Narishige). The stereotaxic coordinates relative to bregma were as follows: cerebral cortex (M1-M2) AP −1.0 mm, ML ±1.0 mm, DV +0.8 mm (advanced to +1.0 mm, then retracted by −0.2 mm); striatum AP −1.0 mm, ML ±1.75 mm, DV +2.75 mm; cerebellum AP +6.5 mm, ML 0 mm, DV 1.8 mm (advanced to +2.0 mm, then retracted by −0.2 mm).

#### Intravenous injection

Mice were anesthetized by intraperitoneal injection of ketamine (100 mg/kg BW) and xylazine (10 mg/kg BW). Anesthetic depth was monitored using the toe-pinch reflex. A 30G needle (08–277; Nipro, Osaka, Japan) was used to access the orbital venous plexus, and 100 μL of AAV solution was slowly injected.

#### Immunohistochemistry

At 21 or 56 days post-injection, mice were transcardially perfused with PBS (pH 7.4) followed by 4% paraformaldehyde in 0.1 M phosphate buffer (PB) (pH 7.4). Brains were post-fixed overnight and then transferred to PBS. Brain slices were prepared using a microtome (VT1200S; Leica, Wetzlar, Germany). Coronal slices (50 μm) were used for the cerebral cortex and striatum, and sagittal slices (50 μm) were used for the cerebellum and whole brain. Slices were incubated with primary antibodies (Table S1) in a blocking solution (2% normal donkey serum, 2% BSA, 0.5% Triton X-100, and 0.05% NaN_3_ in 0.1 M PB) overnight at 4°C. After six washes with PBS, slices were incubated with secondary antibodies (Table S1) in a blocking solution for 3 h at room temperature (24°C–26°C). Slices were then washed six times with PBS and mounted on glass slides with ProLong Diamond Antifade Mountant (P36961; Thermo Fisher Scientific). In Figure 3, staining was performed with two antibodies per slice due to limitations in the number of wavelengths observable in our setup.

#### Imaging analysis

The sagittal sections of the whole brain from intravenously injected mice were acquired using a fluorescence microscope (BZ-X800; Keyence, Osaka, Japan). All other immunostained slices were imaged using a confocal laser-scanning microscope (LSM 800; Carl Zeiss, Oberkochen, Germany). The number of cells was manually counted using z-stack images with ZEISS ZEN software (ZEN 3.6, Carl Zeiss). GFP fluorescence intensity was measured using ImageJ software (https://imagej.net/software/fiji/downloads). Imaging was performed under identical conditions within each experimental group. All images shown are representative.

#### Confocal live imaging in microglia of the motor cortex

Confocal live Ca^2+^ or GFP imaging of microglia was performed in acute cerebral slices from the mouse motor cortex, including primary and secondary motor areas, as described previously,^15^ with some modifications. Coronal slices (250–300 μm thick) of the cerebral cortex containing the motor areas were prepared using a vibroslicer (VT1200S; Leica, Germany) 3–4 weeks after AAV injection into the motor cortex (0.5–5 μL at a titer of 0.4–5 × 10^13^ vg/mL for jGCaMP8s; 1 μL at a titer of 0.1 × 10^13^ vg/mL for GFP). The slices were maintained in artificial cerebrospinal fluid (ACSF) containing (in mM): 125 NaCl, 2.5 KCl, 2 CaCl_2_, 1 MgCl_2_, 1.25 NaH_2_PO_4_, 26 NaHCO_3_, and 10 D-glucose, bubbled with 95% O_2_ and 5% CO_2_ at room temperature for over one hour before recording. Image acquisition, processing, and analysis were performed using Andor iQ3 (Andor), NIH ImageJ, Igor Pro9 (WaveMetrics) with Neuromatic (http://www.neuromatic.thinkrandom.com),^37^ and custom-written programs by NH.

The ‘jGCaMP8s’ sensor protein is a fast and sensitive genetically encoded Ca^2+^ indicator that increases fluorescence upon an elevation in intracellular Ca^2+^ concentration.^22^ To monitor Ca^2+^ signals in jGCaMP8s-expressing microglia in brain slices, confocal fluorescence images were acquired every 2 s using a water-cooled EM-CCD camera (200 ms exposure time, 512 × 512 pixels; iXon3 DU-897E-CS0-#BV-500; Andor, Belfast, Northern Ireland), a 40× water immersion objective (LUMPLFLN 40XW; Olympus, Tokyo, Japan), and a high-speed spinning-disk confocal unit (CSU-X1; Yokogawa Electric, Tokyo, Japan) attached to an upright microscope (BX51WI; Olympus, Tokyo, Japan). A blue laser light (488 nm; Stradus 488-50; VORTRAN, Sacramento, CA) was used for excitation, and emitted fluorescence was collected through a 500–550 nm band-pass filter. During recordings, cortical slices were continuously perfused with ACSF bath solution at room temperature. ATP (100 μM) dissolved in ACSF was bath-applied for 1.5–4 min via a gravity-fed bath-application device to elicit intracellular Ca^2+^ increases and process movement in microglia. Image drift (translation drift) during recordings was corrected using the Image Stabilizer plugin (http://www.cs.cmu.edu/∼kangli/code/Image_Stabilizer.html) or the Template Matching and Slice Alignment plugin (https://sites.google.com/site/qingzongtseng/template-matching-ij-plugin#downloads) in ImageJ.

GCaMP fluorescence intensity at time t (F_t_) in each pixel was background-subtracted, and Ca^2+^-dependent relative changes in fluorescence were measured by calculating ΔF/F_basal_, where F_basal_ is the basal fluorescence intensity averaged during pre-stimulus frames (i.e., more than 10 frames before ATP application) and ΔF = F_t_ - F_basal_. Background fluorescence was measured from regions devoid of cellular structures in the same frame. Mean ΔF/F_basal_ values were calculated for each region of interest (ROI) in each frame. ROIs were placed on GCaMP-positive cellular structures. Because we could not determine whether multiple neighboring ROIs in a frame corresponded to the same cell, each ROI was referred to as a microglial cellular compartment. Ca^2+^ imaging data were collected from 15 different fields of view, each separated by more than 200 μm, in eight slices from five mice. Considering the coverage of microglial process territories (convex hull areas ranging from 800 to 3800 μm^2^, corresponding to circles with diameters of several tens of micrometers),^25^ we estimate that the Ca^2+^ imaging data originated from more than 15 microglia. To quantify ATP-induced Ca^2+^ signals in GCaMP-positive cells, the peak amplitude of ΔF/F_basal_ was measured within a 100-s time window after the onset of ATP application.

To capture live morphological dynamics in GFP-positive microglia, confocal GFP fluorescence images were acquired every 2 s under the same camera settings as for GCaMP imaging (single focal plane imaging). This method often missed out-of-focus microglial processes (Figure S4, Single focal plane) due to the complex three-dimensional structure of microglia.^25^ To overcome this limitation, z stack images were acquired at multiple focal planes, covering the entire microglial structure with a depth range of 40–73 μm. These z-stacks were obtained at each time point every 6–9 s through rapid z-axis positioning of the objective lens (z-step size: 1.65–1.94 μm) using a piezo objective scanner (PFM450E, Thorlabs). Maximum intensity projections of the z-stacks on the xy plane were generated to create 2D time-lapse images. The signal-to-noise ratio of the GFP signal was significantly higher than that of the GCaMP signal under these experimental conditions. Image drift during GFP imaging was corrected using the same method as in Ca^2+^ imaging.

### Quantification and statistical analysis

GraphPad PRISM (version 9; GraphPad Software, San Diego, CA, USA) was used for statistical analyses and data visualization. The statistical details of experiments are found in Results or each figure legends. Specificity, efficiency, and fluorescence intensity data were presented as box-and-whisker plots. The box-and-whisker plots depict the median (centerlines), 25th and 75th percentiles (bounds of the box), and minimum/maximum values (whiskers). Statistical significance for specificity and efficiency was determined using unpaired t-tests or Bonferroni’s multiple comparisons test after one-way ANOVA. Statistical significance for fluorescence intensity was determined using unpaired t-tests (Figure 2F). Comparisons of fluorescence intensity at 3 weeks versus 2 months post-injection were presented as cumulative plot distributions and analyzed with the Kolmogorov-Smirnov test (Figures 5E and 5F). Unless otherwise indicated, data values presented in the main text are expressed as mean ± standard deviation (SD).

## Supplemental information

Document S1. Figures S1–S8 and Table S1

Document S2. Article plus supplemental information
