# Supplementary material for: AAV vectors for specific and efficient gene expression in microglia
Source: Cell Rep Methods. 2025 Jul 30;5(8):101116. doi: 10.1016/j.crmeth.2025.101116 (PMC12461629; doi:10.1016/j.crmeth.2025.101116)
Supplement: Document S1. Figures S1–S8 and Table S1 [file mmc1.pdf]

Cell Reports Methods, Volume *5*

Supplemental information

AAV vectors for specific and efficient gene expression in microglia

Ryo Aoki, Ayumu Konno, Nobutake Hosoi, Hayato Kawabata, and Hirokazu Hirai

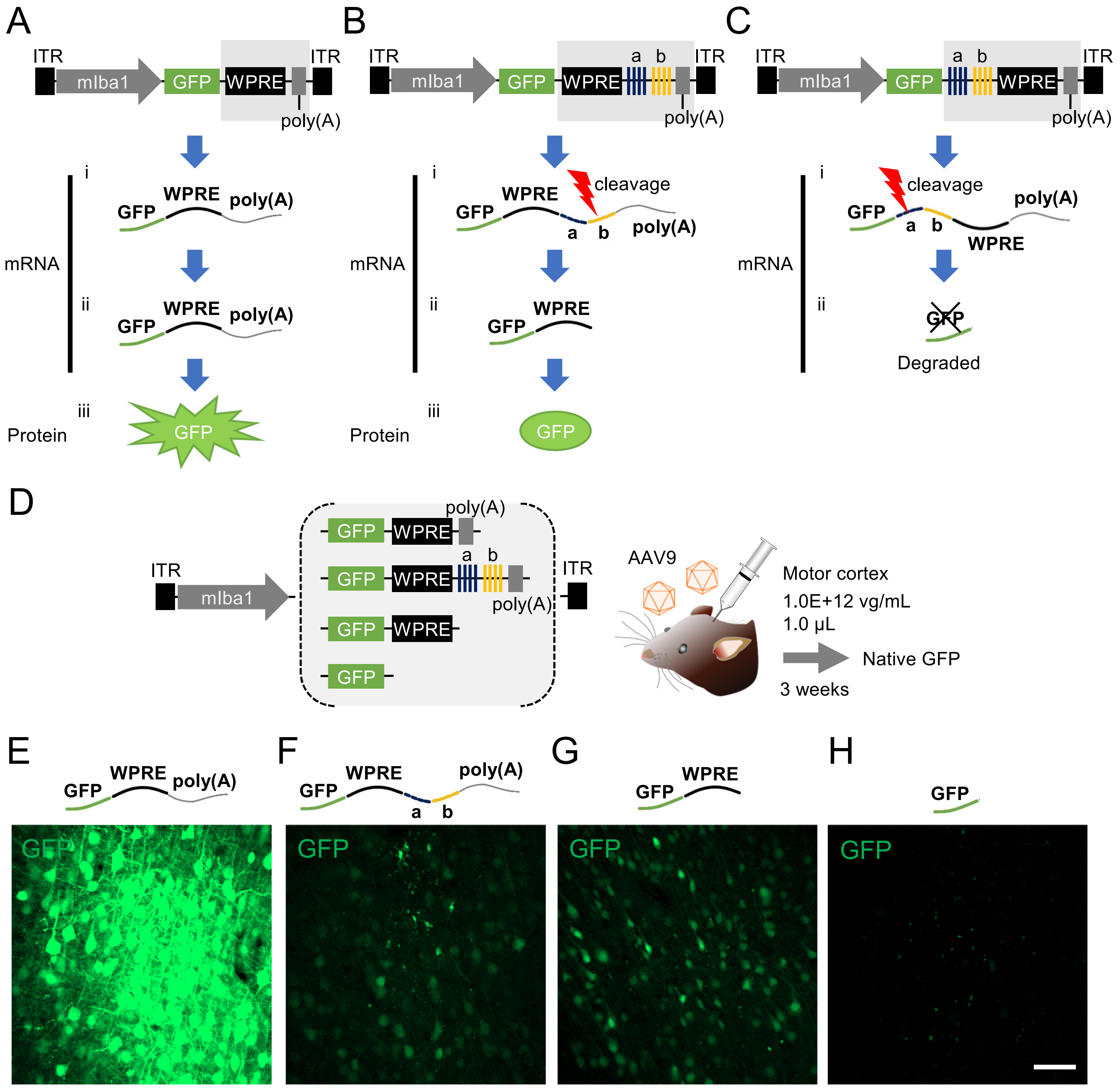

Figure S1. A hypothetical model and experiments demonstrating that GFP expression is suppressed when miR.T is located on the 5' side of WPRE, related to Figure 2
(A) AAV.mIba1.GFP.WPRE.poly(A) produces mRNA consisting of GFP, WPRE, and poly(A) signal, resulting in strong GFP expression. **(B)** Insertion of miR.Ts on the 3' side of WPRE produces mRNA containing GFP, WPRE, miR.Ts, and poly(A) signal. This mRNA is cleaved at the miR.Ts by RNA interference in neurons. The processed GFP and WPRE mRNA may still be translated into GFP, although less efficiently than without miR.Ts (as shown in (A)). **(C)** Insertion of miR.Ts on the 5' side of WPRE produces mRNA containing GFP, miR.Ts, WPRE, and poly(A) signal. This mRNA is likely processed into mRNA containing only GFP, which may lead to its degradation. **(D)** Experimental design to test this hypothesis. AAV.mIba1.GFP with or without WPRE, miR.T, and poly(A) signal was prepared as indicated. Mice received cerebral injections of one of the AAVs and were euthanized three weeks later to observe native GFP expression. **(E-H)** Confocal micrographs of cerebral sections from mice injected with AAVs as shown in (D). The mRNA transcribed from the injected AAV is illustrated above each panel. Scale bar: 50 µm.

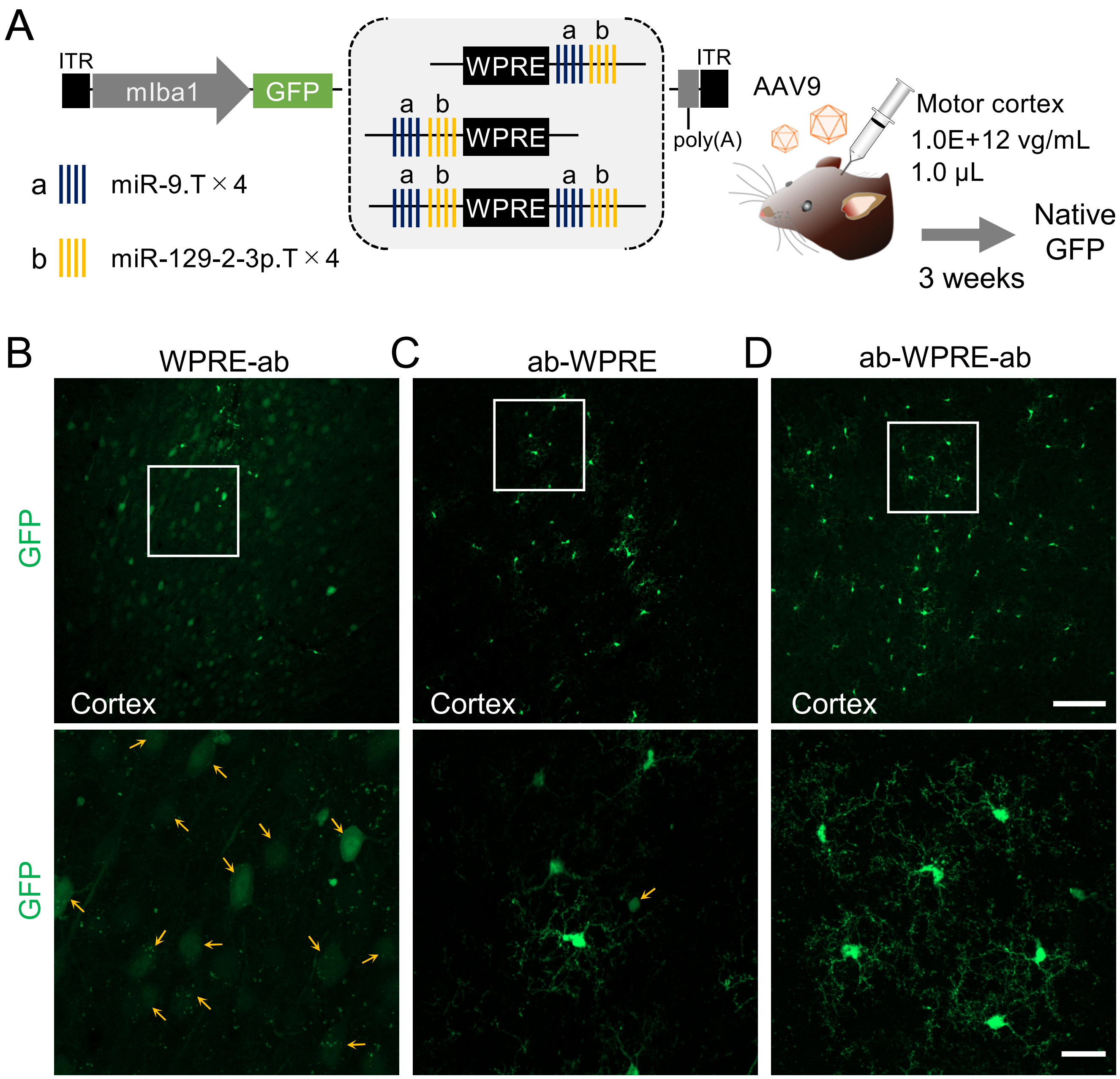

Figure S2. Native GFP fluorescence in cortical microglia of mice injected with AAV9.mIba1.GFP.WPRE, with miR.T on the 3', 5', or both sides of WPRE, related to Figure 2

(A) Schematic showing three AAV genomes with miR.T sequences positioned downstream, upstream, or on both sides of WPRE. One of these AAVs was injected into the motor cortex at the indicated doses, and the cerebral cortex was sectioned three weeks after injection to observe native GFP fluorescence. **(B-D)** Native GFP fluorescence images of the cerebral cortex. The boxed areas in the lower magnification images are enlarged and shown below. Yellow arrows indicate cells morphologically distinct from microglia. Scale bars: 100 μm (upper right) and 20 μm (lower right).

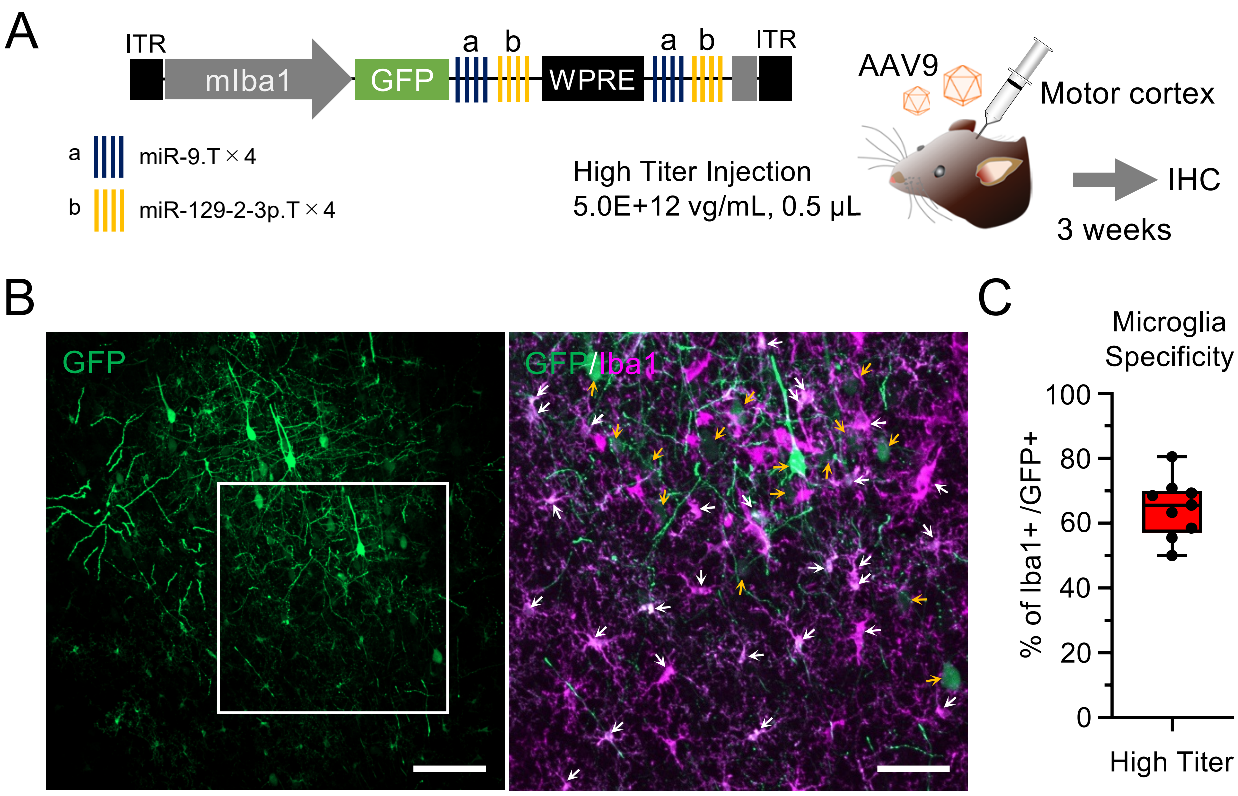

Figure S3. High-titer injection of AAV-mIba1-GFP-ab-WPRE-ab leads to off-target GFP expression in neurons despite robust microglial transduction, related to Figure 2

(A) Schematic illustration of the AAV genome construct used: AAV9 carrying mIba1.GFP.ab-WPRE-ab was produced and injected at high titer (5.0E+12 vg/mL, 0.5 μL) into the mouse motor cortex. Mice were euthanized three weeks after injection for immunohistochemistry (IHC). (B) Confocal images of the cerebral cortex. Left panel shows a low-magnification image of GFP immunostaining; right panel shows a high-magnification image of GFP and Iba1 immunostaining, corresponding to the boxed region in the low-magnification image. White arrows indicate GFP^+ microglia; yellow arrows indicate GFP^+ neurons. Scale bars: 100 μm (low magnification), 50 μm (high magnification). (C) Summary graph of microglial specificity (n = 9 hemispheres). Box-and-whisker plots show the median (centerline), interquartile range (box), and minimum/maximum values (whiskers).

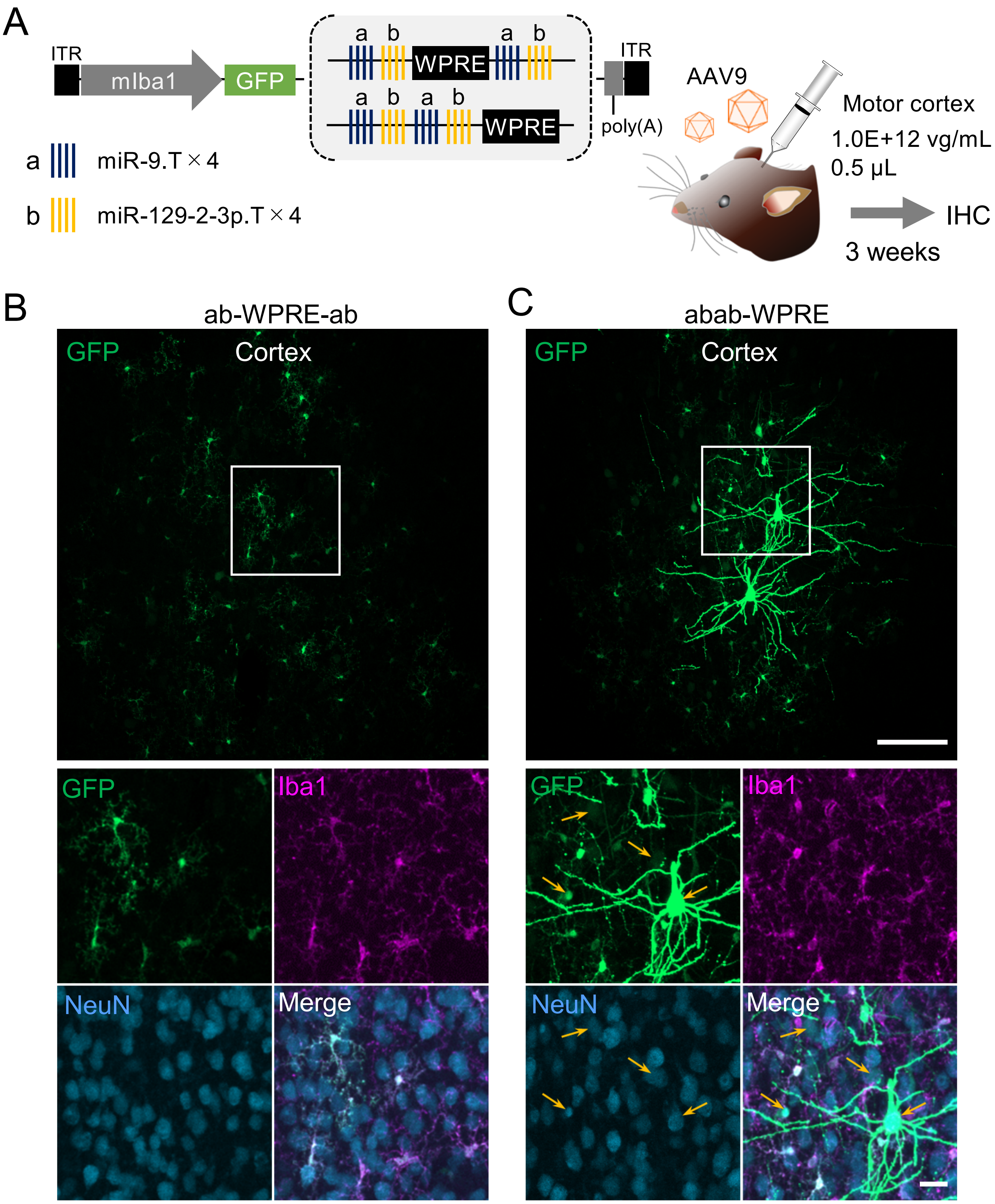

Figure S4. Placing miR.Ts on both sides of the WPRE is crucial for increasing the specificity of GFP expression in microglia, related to Figure 2

(A) AAV.mIba1.GFP with two sets of miR.Ts on each side of WPRE, or with four sets of miR.Ts only upstream of WPRE, was injected into the mouse cerebral cortex at the indicated doses. **(B, C)** Confocal microscopy of cortical sections three weeks after injection of AAV.mIba1.GFP with ab-WPRE-ab (B) or abab-WPRE (C). The boxed areas in the low-magnification images are shown enlarged below. Yellow arrows indicate NeuN- and GFP-double positive neurons. Scale bars: 100 μm (upper right) and 20 μm (bottom right).

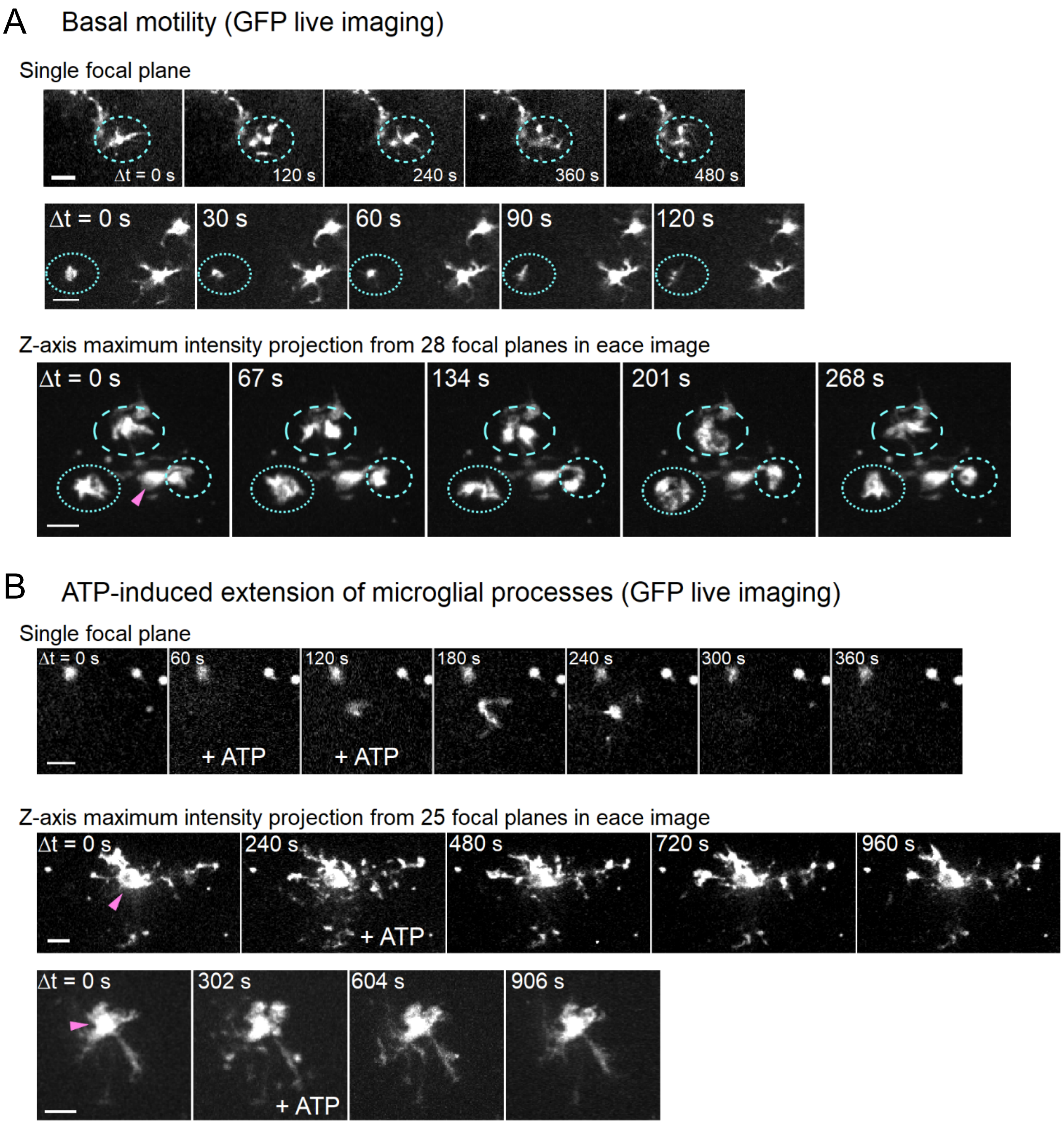

Figure S5. Monitoring microglial motility in the mouse motor cortex using our microglia-selective AAV-mediated gene expression method, related to Figure 6

(A, B) Time-lapse GFP fluorescent images with time points indicated, showing basal movements of microglial processes in (A) and ATP-induced process extension in (B). Frames during the bath application of 100 μM ATP (3–4.5 minutes) are labeled as '+ ATP'. Blue circles highlight areas with pronounced basal motility, and magenta arrowheads indicate the putative cell bodies of microglia in maximum intensity projection images of z-stacks. Each row of time-lapse images in (A) and (B) represents different microglia. Scale bars: 10 μm. See also Video S2-S9.

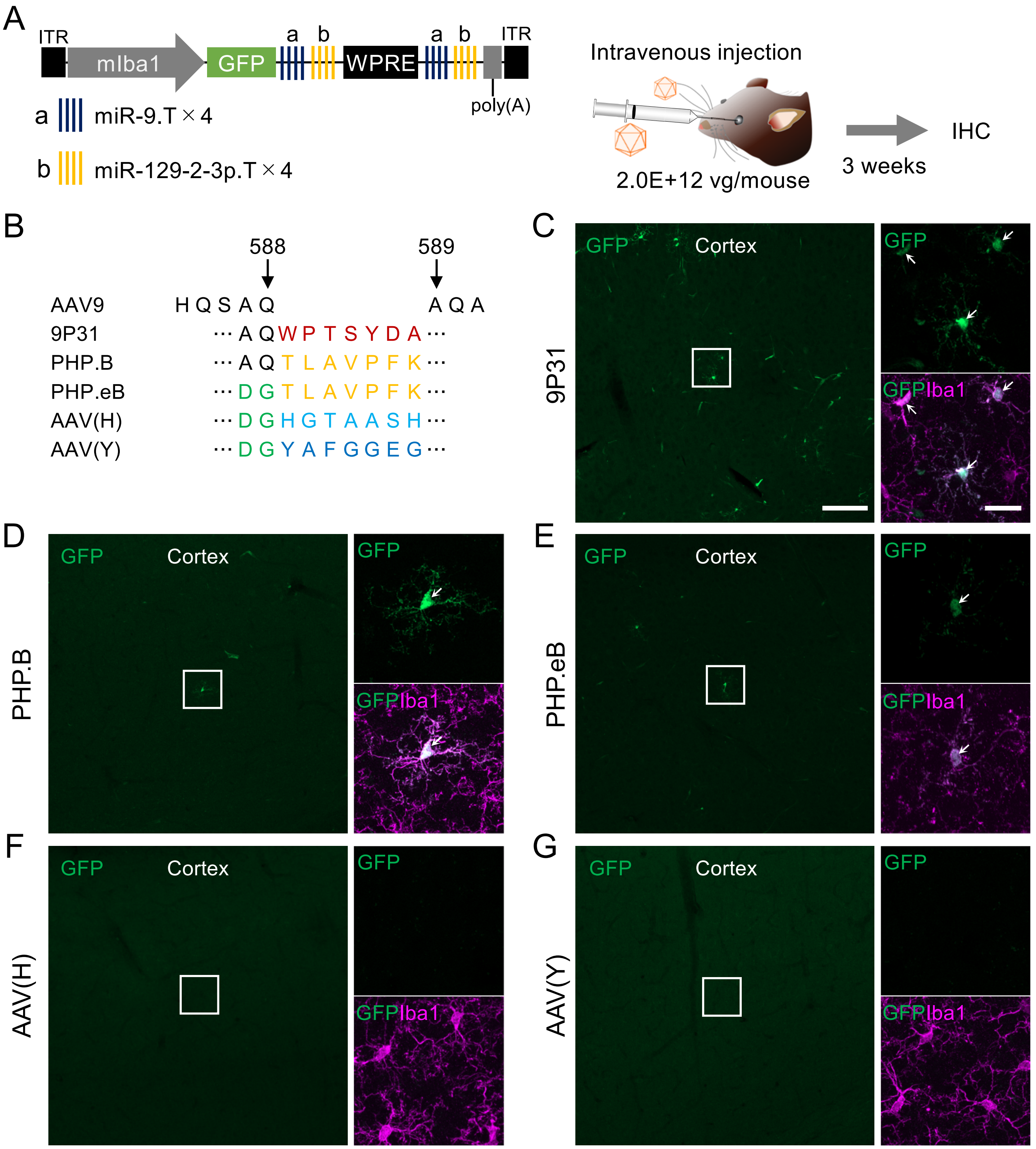

Figure S6. Comparison of five BBB-permeable capsid mutants for gene expression in microglia upon intravenous administration, related to Figure 7

(A) Five different BBB-penetrating capsid vectors containing mIba1.GFP.ab-WPRE-ab.poly(A) were injected intravenously into mice via the orbital plexus. **(B)** The seven amino acid insertions between 588Q and 589A of the AAV9 capsid in the BBB-penetrating capsid variants (including additional flanking mutations) are presented alongside the parental AAV9 sequence. **(C–G)** Confocal microscopy images of cortical sections from mice injected intravenously with microglia-targeting, BBB-permeable AAV vectors. The BBB-penetrating capsid mutants used are shown at the left side of each panel. Boxed areas are enlarged and displayed on the right sides. Arrows indicate microglia double immunostained for GFP and Iba1. Scale bars in panel (C): 100 μm (left) and 20 μm (lower right).

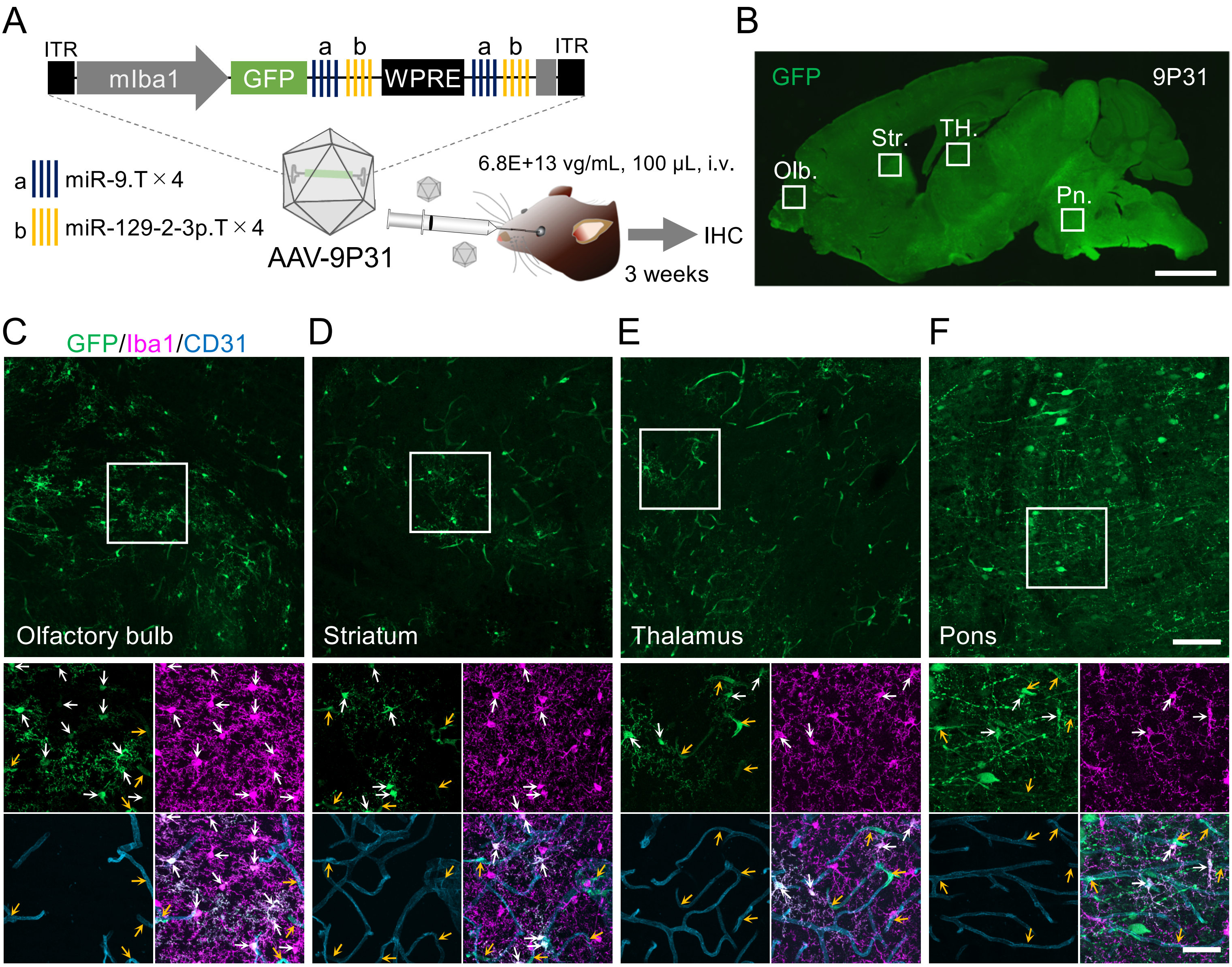

Figure S7. GFP expression in microglia and brain microvascular endothelial cells across various brain regions following intravenous injection of microglia-targeting AAV-9P31 vectors, related to Figure 7
(A) High dose of AAV-9P31.mIba1.GFP.ab-WPRE-ab.poly(A) (6.8E+13 vg/mL, 100 μL) was intravenously injected. Sagittal brain sections were prepared, and GFP-expressing cell types were examined by immunohistochemistry (IHC). **(B)** GFP immunolabeling image of a sagittal brain section from a mouse intravenously injected with AAV-9P31. Scale bar, 2mm. **(C–F)** Upper panels: Enlarged GFP immunofluorescent images of the boxed regions in (B): thalamus (C), olfactory bulb (D), striatum (E), and pons (F). Lower panels: Further enlarged immunohistochemical images of the boxed areas in the respective upper panels. Microglia and vascular endothelial cells were immunolabeled with anti-Iba1 (magenta) and anti-CD31 (blue) antibodies, respectively. White and yellow arrows indicate GFP-positive microglia and vascular endothelial cells, respectively. Scale bars in the upper and lower panels of (F): 100 μm and 40 μm, respectively.

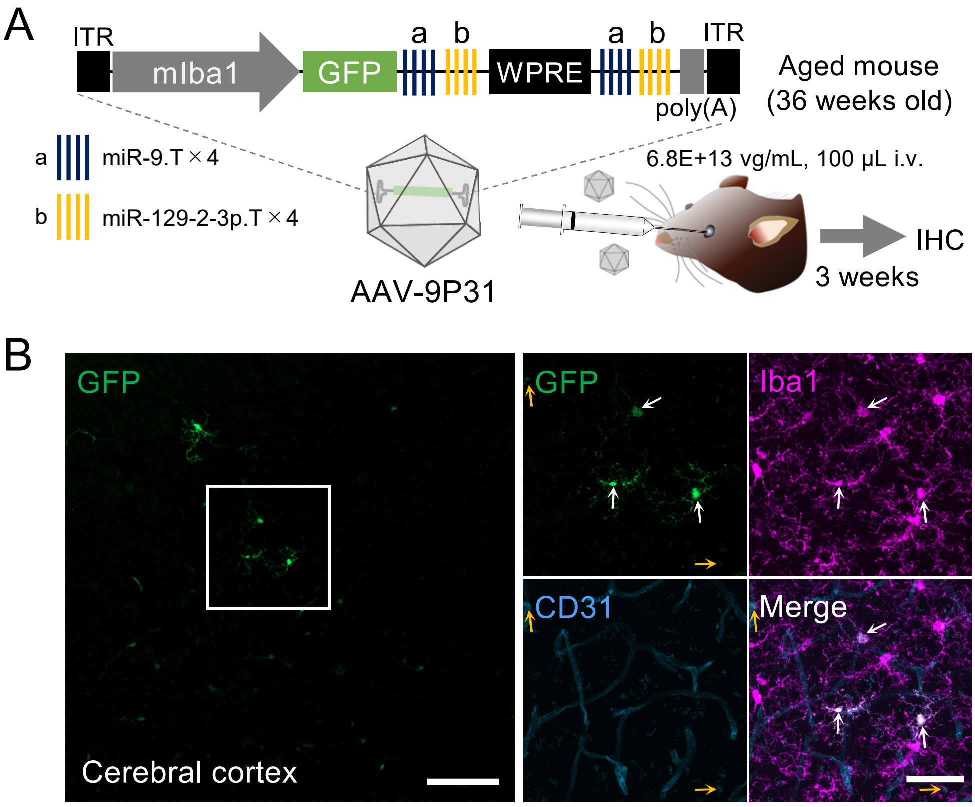

Figure S8. GFP labeling of microglia in a middle-aged mouse following intravenous injection of AAV-9P31, related to Figure 7

(A) A microglia-targeting, BBB-penetrant vector (AAV-9P31.mIba1.GFP.ab-WPRE-ab) was intravenously injected into a 36-week-old C57BL/6J mouse via the orbital venous plexus (6.8E+13 vg/mL, 100 µL). The mouse was euthanized three weeks post-injection, and the brain was processed for immunohistochemistry (IHC). **(B)** The left panel shows a low-magnification fluorescent image of GFP immunostaining in the cerebral cortex. The four right panels show higher magnification views of the boxed region in the left panel, illustrating immunolabeling for GFP, Iba1, and CD31. White and yellow arrows indicate GFP-expressing microglia and vascular endothelial cells, respectively. Scale bars: 100 µm (left), 40 µm (right).

Table S1. Antibody list, related to Figure 1-5, 7, and Figure S3-4, S6-8

Primary antibody

No.	Antibody	Host	Monoclonal/ Polyclonal	Dilution ratio	Source	Identifier
1	anti-GFP	Rat	Mono	×1000	Nacalai	04404-84
2	anti-Iba1	Rabbit	Poly	×500	Wako	019-19741
3	anti-NeuN	Mouse	Mono	×1000	Millipore	MAB377
4	anti-S100β	Rabbit	Poly	×200	Nittobo Medical	S100β-Rb-Af1000
5	anti-Olig2	Mouse	Mono	×500	Sigma-Aldrich	MABN50
6	anti-GFP	Goat	Poly	×200	Nittobo Medical	GFP-Go-Af1480
7	anti-CD31	Rat	Poly	×100	BD Biosciences	550274

Secondary antibody (Alexa Fluor Plus)

No.	Antibody	Wavelength	Host	Monoclonal/ Polyclonal	Dilution ratio	Source	Identifier
1	anti-Rat IgG	488	Donkey	Poly	×2000	Thermo Fisher	A48269
2	anti-Rat IgG	647	Donkey	Poly	×2000	Thermo Fisher	A48272
3	anti-Rabbit IgG	555	Donkey	Poly	×2000	Thermo Fisher	A32794
4	anti-Mouse IgG	647	Donkey	Poly	×2000	Thermo Fisher	A32787
5	anti-Goat IgG	488	Donkey	Poly	×2000	Thermo Fisher	A32814

Antibodies used in each figure

Figure	Primary	Secondary
Fig. 1B, C	1, 2, 3	1, 3, 4
Fig. 2B, C	1, 2, 3	1, 3, 4
Fig. 3B	1, 2, 3	1, 3, 4
Fig. 3C (Iba1, NeuN)	1, 2, 3	1, 3, 4
Fig, 3C (S100β, Olig2)	1, 4, 5	1, 3, 4
Fig. 4B, C	1, 2, 3	1, 3, 4
Fig. 5B, C	1, 2, 3	1, 3, 4
Fig. 7B, C	2, 6, 7	2, 3, 5
Fig. 7D (Cell count for Exclude CD31+)	2, 6, 7	2, 3, 5
Fig. 7E, F (Cell count)	1, 2, 3	1, 3, 4
Fig. S3B	1, 2, 3	1, 3, 4
Fig. S4B, C	1, 2, 3	1, 3, 4
Fig. S6C-G	1, 2, 3	1, 3, 4
Fig. S7B-F	2, 6, 7	2, 3, 5
Fig. S8B	2, 6, 7	2, 3, 5
